# The Roles of APC and Axin Derived from Experimental and Theoretical Analysis of the Wnt Pathway

**DOI:** 10.1371/journal.pbio.0000010

**Published:** 2003-10-13

**Authors:** Ethan Lee, Adrian Salic, Roland Krüger, Reinhart Heinrich, Marc W Kirschner

**Affiliations:** **1**Department of Cell Biology, Harvard Medical SchoolBoston, MassachusettsUnited States of America; **2**Department of Theoretical Biophysics, Institute of BiologyHumboldt University Berlin, BerlinGermany; **3**Department of Cell and Developmental Biology, Vanderbilt University Medical CenterNashville, TennesseeUnited States of America

## Abstract

Wnt signaling plays an important role in both oncogenesis and development. Activation of the Wnt pathway results in stabilization of the transcriptional coactivator β-catenin. Recent studies have demonstrated that axin, which coordinates β-catenin degradation, is itself degraded. Although the key molecules required for transducing a Wnt signal have been identified, a quantitative understanding of this pathway has been lacking. We have developed a mathematical model for the canonical Wnt pathway that describes the interactions among the core components: Wnt, Frizzled, Dishevelled, GSK3β, APC, axin, β-catenin, and TCF. Using a system of differential equations, the model incorporates the kinetics of protein–protein interactions, protein synthesis/degradation, and phosphorylation/dephosphorylation. We initially defined a reference state of kinetic, thermodynamic, and flux data from experiments using Xenopus extracts. Predictions based on the analysis of the reference state were used iteratively to develop a more refined model from which we analyzed the effects of prolonged and transient Wnt stimulation on β-catenin and axin turnover. We predict several unusual features of the Wnt pathway, some of which we tested experimentally. An insight from our model, which we confirmed experimentally, is that the two scaffold proteins axin and APC promote the formation of degradation complexes in very different ways. We can also explain the importance of axin degradation in amplifying and sharpening the Wnt signal, and we show that the dependence of axin degradation on APC is an essential part of an unappreciated regulatory loop that prevents the accumulation of β-catenin at decreased APC concentrations. By applying control analysis to our mathematical model, we demonstrate the modular design, sensitivity, and robustness of the Wnt pathway and derive an explicit expression for tumor suppression and oncogenicity.

## Abstract 2

Wnt signaling is important in both oncogenesis and development. Mathematical modeling predicts several unusual features of the Wnt pathway, some of which are tested experimentally

## Introduction

Considerable effort employing biochemistry, genetics, and pharmacology has been invested in identifying the web of interactions that characterize signal transduction pathways in metazoan organisms. Several conclusions can be drawn from these efforts. Despite the large number of receptors, ligands, and downstream targets, the number of signal transduction pathways in metazoans is relatively small, arguably less than 20 (Gerhart 1999). This limited diversity occurs despite large numbers of different organisms, cell types, states of growth, and differentiation, as well as sexual dimorphism in biology. Remarkably, these pathways are highly conserved, some among all eukaryotes, most among all metazoans. Whereas signaling pathways differ in detail, it is not clear whether these differences are functionally significant. Conservation in the face of diversity of function raises the question of whether the behaviors of the pathway are in reality as similar as they seem when one compares more quantitative aspects of the signals and responses, such as amplitude, duration, and flux (Heinrich et al. 2002). Finally, the structure and design of the pathways are themselves a mystery. Is the structure of these conserved pathways so deeply embedded in other conserved process that it is difficult to change any interaction, or does conservation imply continuous selection for function (Gerhart and Kirschner 1997)?

Many of these questions require a more quantitative understanding of the behavior of signaling pathways. Such information is rarely available. Most mathematical models have to be satisfied with a general conceptual understanding and are seldom testable, as most parameters must be assumed or inferred. It is partly for this reason that such theoretical efforts up to now have had limited impact on experimentalists, who prefer powerful qualitative tools to construct logical and formal models of pathway structures. Mathematical modeling is more advanced for metabolic networks, where the pathways have been known for more than a half-century and where more kinetic data have been available, including more recent data on in vivo dynamics (Heinrich and Schuster 1996). To develop a better quantitative understanding of a signal transduction pathway, we have recreated a more accessible system for biochemical study. The Wnt signaling pathway downstream of its immediate cytoplasmic mediator, Dishevelled (Dsh), can be reconstituted in frog egg extracts. The readout of the pathway is the rate of degradation of the transcriptional coactivator, β-catenin. We chose the Wnt pathway because it is active in the early Xenopus embryo, it is widely used in many different contexts in development, and it is also very important in human cancer. Although the design features of the Wnt pathway are highly conserved in evolution, it is not clear what purposes those features serve. This paper is in part an answer to that question.

The pivotal player in Wnt signaling is the scaffold protein axin, which is required for the constitutive degradation of β-catenin. Axin coordinates the assembly of a large complex that includes the glycogen synthase kinase 3β (GSK3β); another scaffold protein, adenomatous polyposis coli (APC); and the negative regulators Dsh and GSK3β-binding protein (glycogen synthase kinase-binding protein [GBP]/Frat). Binding of Wnt to its receptor, Frizzled, activates Dsh through an as-yet-unknown process. In the absence of Wnt, GSK3β bound to axin phosphorylates β-catenin bound to both axin and APC. Phosphorylated β-catenin is a substrate for ubiquitination and subsequent degradation through the F-box protein β-TRCP, which is part of an SCF ubiquitin ligase complex. In the presence of the Wnt signal, the activated Dsh protein binds to axin and, through bound GBP, inhibits β-catenin phosphorylation; this inhibits its ubiquitination and consequent degradation. The buildup in β-catenin in the presence of a Wnt signal leads to transcription of specific genes.

Numerous questions arise from this general model. Why are two scaffold proteins, APC and axin, both necessary? Do their roles differ? Recently it has been discovered that axin, like β-catenin, is an unstable protein (Yamamoto et al. 1999; Tolwinski et al. 2003). In recent work (unpublished data), we have further described the conditions under which axin is unstable. We ask here what role axin instability plays in the behavior of the Wnt pathway and in the responsiveness of the pathway to the Wnt signal? Beyond these mechanistic questions are important biological ones that lie beyond the scope of this work but that may be raised by some of the findings here. For example, mutations in APC seem to play a particularly important role in colorectal cancer; is the peculiar sensitivity to APC mutations in the colonic epithelium understandable in terms of how the pathway performs in that tissue? Similarly, GSK3β is also essential but not commonly mutated in colorectal cancer; why is that the case? No one has purified a discrete complex containing all the major players in the Wnt pathway arrayed on the axin–APC scaffolds, suggesting that the pathway might be affected by nonproductive titration of components by subcomplexes. If this is a problem, how is it avoided? The Wnt pathway is likely present in all cell types, and yet several of its constituents are used in other pathways; how is crosstalk or interference in other pathways avoided? As we set out to produce a realistic kinetic model of the Wnt pathway, we encountered other questions. For example, quantitative measurements led to the unusual finding that axin is present at very low concentrations. Is there a satisfactory explanation of this fact and of other, previously unexplained, features of the pathway? We are aware that some of the answers to specific questions could lie in unknown components or unknown interactions among known components. We were under no illusions that we could accommodate all known interactions in a model at this time or that we already knew all we need to know to construct such a model. For this reason we have asked a more modest question—whether the properties of the core pathway, as presently understood, can provide insight into important questions not yet answered or not yet clearly raised.

To answer such questions, we collected what we initially thought were sufficient quantitative data on rates, affinities, and fluxes to derive a reference state model of the Wnt pathway in this system. The provisional reference state model reflected most of the known properties of the system, but from this model we identified several rates and affinity constants whose values were critical to the behavior of the model. We then went back and measured these. Thus this paper contains a reference model, a large number of experimental measurements needed to define this model, theoretical predictions, and experimental tests of those predictions, where possible. A general test of the validity of the model is its predictive ability under a wide range of conditions. From this analysis, several unexpected properties emerged with significance for understanding the biological function of the Wnt pathway.

## Results

### A Proposed Kinetic Pathway

**Figure 1:**
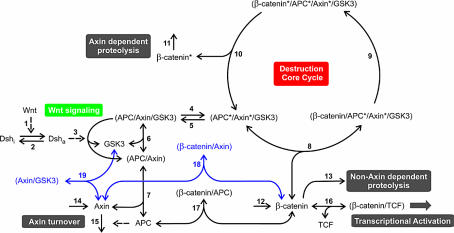
Reaction Scheme for Wnt Signaling The reaction steps of the Wnt pathway are numbered 1 to 19. Protein complexes are denoted by the names of their components, separated by a slash and enclosed in brackets. Phosphorylated components are marked by an asterisk. Single-headed solid arrows characterize reactions taking place only in the indicated direction. Double-headed arrows denote binding equilibria. Blue arrows mark reactions that have only been taken into account when studying the effect of high axin concentrations. Broken arrows represent activation of Dsh by the Wnt ligand (step 1), Dsh-mediated initiation of the release of GSK3β from the destruction complex (step 3), and APC-mediated degradation of axin (step 15). The broken arrows indicate that the components mediate but do not participate stoichiometrically in the reaction scheme. The irreversible reactions 2, 4, 5, 9–11, and 13 are unimolecular, and reactions 6, 7, 8, 16, and 17 are reversible binding steps. The individual reactions and their role in the Wnt pathway are explained in the text.

The model was based on the reaction scheme shown in Figure 1. A few steps are labeled, such as the synthesis of axin and β-catenin, the degradation of axin, the axin-independent (basal) and axin-dependent degradation of β-catenin, as well as the critical cycle involved in the phosphorylation of β-catenin for degradation (Destruction Core Cycle). The output is the formation of the β-catenin/T-cell factor (TCF) complex and the input is the Wnt signal. Although many proteins interact with the Wnt pathway, we have focused only on core components known to be necessary for mediating a Wnt signal in most contexts. These core proteins include GSK3β, protein phosphatase 2A (PP2A), β-catenin, APC, axin, Dsh, TCF, and Wnt. The reactions incorporated into our model include protein synthesis/degradation, protein phosphorylation/dephosphorylation, and the assembly/disassembly of protein complexes (Figure 1, solid arrows). Reactions mediated by proteins that activate a process are represented with broken arrows: (1) activation of Dsh by Wnt (step 1), (2) activation of the release of GSK3β from APC/axin/GSK3β by Dsh (step 3), and (3) activation of APC-dependent axin degradation (step 15). The reactions and components in blue are concerned with additional features of the pathway, as discussed below.

The centerpiece of the model is the formation of the unstable core complexes involved in β-catenin phosphorylation and subsequent destruction. In addition to β-catenin, this set of complexes contains GSK3β and the scaffold proteins APC and axin. The complexes assemble in several steps: (1) binding of axin to APC (forward reaction of step 7); (2) binding of GSK3β (forward reaction of step 6); (3) phosphorylation of axin and APC by GSK3β (step 4). Dephosphorylation of the core complex (step 5) is mediated by PP2A. The first step in β-catenin degradation is its binding to APC*/axin*/GSK3β (step 8), after which it is phosphorylated by GSK3β (step 9) and released from the complex (step 10). Our model assumes that the phosphorylation of β-catenin by GSK3β is negligible in the absence of axin. Indeed, recent work indicates that axin stimulates the phosphorylation of β-catenin by GSK3β at least 24,000-fold (Dajani et al. 2003). Free, phosphorylated β-catenin is rapidly polyubiquitinated and degraded by the SCF complex and the proteasome, respectively (step 11).

The dynamic properties of the model, such as the flux through the pathway, are also affected by binding of β-catenin to other partners, such as TCF (step 16) and free APC (step 17). In special cases (high axin concentrations), the flux through the system is affected by the binding of axin to GSK3β (step 19) as well as β-catenin (step 18). We have previously shown experimentally that TCF reduces the rate of β-catenin degradation (Lee et al. 2001). Turnover of β-catenin (steps 11, 12, and 13) and axin (steps 14 and 15) are included in our model, but since biochemical experiments in Xenopus egg extracts indicate that the turnover of GSK3β, Dsh, and TCF is relatively slow (no detectable degradation after 3 h at room temperature; unpublished data), the synthesis and degradation of these proteins are not explicitly modeled. The activation of the pathway in vivo, which results in the stabilization of β-catenin, is initiated by binding of Wnt ligands to Frizzled receptors and the subsequent transition of Dsh from its inactive form (Dsh_i_) to its active form (Dsh_a_). Since these events are still poorly defined, both processes have been combined in step 1. Interaction of Dsh_a_ with the nonphosphorylated complex APC/axin/GSK3β (step 3) activates the release of GSK3β . This latter process requires the activity of the GBP/Frat (not shown on our diagram). Deactivation of Dsh_a_ occurs through an as-yet-unidentified mechanism (step 2).

### Analytical Description

The mathematical analysis is based on a series of balance equations that describe the concentrations and complexes of proteins in the Wnt pathway, as depicted in Figure 1. The set of variables and the set of 15 differential equations we obtained are given in Table S1, and Dataset S1, respectively (Equations [A-1]–[A-15]). Stimulation of the pathway by Wnt is described by a time-dependent function, *Wnt(t)*. Since Dsh, TCF, and GSK3β are degraded very slowly, we assume that their concentrations remain constant throughout the timecourse of a Wnt signaling event. The conservation equations for Dsh, TCF, and GSK3β are as follows:

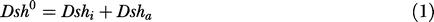


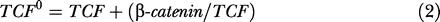


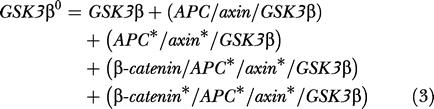
Symbols with the superscript "0" denote total concentrations.

Since the concentration of axin is very low (see below) compared to the concentration of GSK3β, we replaced Equation (3) with the simple relationship *GSK3β* ^0^ = *GSK3β*. Similarly, we omitted the concentration of complexes containing axin in the conservation relationship for APC, which leads to the following equation:

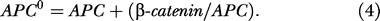



We will, however, take into account the contribution of axin-containing complexes for GSK3β and APC conservation equations when we later consider the effect of large increases in axin concentration.

The simplest possible equation was chosen to describe the kinetics of each individual reaction. Synthesis of β-catenin and axin are described by constant rates **ν** *_i_*. Unimolecular reactions are assumed to be irreversible and are described by linear rate equations **ν** *_i_* = *k_i_* · *X_j_*, where *k_i_* denotes the first-order rate constant and *X_j_* denotes the concentration of the reactants. Reversible binding steps (double-headed arrows in Figure 1) are described by the equation **ν** *_i_* = *k_+i_X_j_Y_l_* − *k_−i_*(*X_j_* **.** *Y_l_*), where *X_j_* and *Y_l_* denote the concentrations of the binding partners and (*X_j_* **.** *Y_l_*) the concentration of their complex. The Dsh-mediated release of GSK3β from the destruction complex is described by an irreversible reaction that is bimolecular in the concentrations of Dsh and the degradation complex. The model is simplified by assuming that the reversible binding steps between axin, β-catenin, APC, and TCF are very fast, such that the corresponding protein complexes are in rapid equilibrium, so that only the dissociation constants *K_i_* = *k*_−*i*_/*k*_+*i*_ are considered in the kinetic description of these steps. The conservation equations and the binding equilibria reduce the number of independent dynamic variables. Accordingly, the original set of 15 differential equations is transformed into a set of only seven ordinary differential equations coupled to four conservation equations and four relationships for binding equilibria. For a detailed mathematical description of the model and the procedure for reducing the number of systems variables, see Dataset S1.

### Experimental Evaluation of the Reference and Stimulated States

We define the reference state as the absence of Wnt signaling (*Wnt* = 0). In this unstimulated stationary state, Dsh is inactive and does not affect the degradation complex. β-Catenin concentration is kept low by continuous phosphorylation and degradation. The reference state can be characterized by the special values for its rate constants, its equilibrium constants, and its conservation quantities. If one can obtain values for all of these system parameters, the model equations should allow for a straightforward calculation of the variables in the reference state. Currently, we have experimental data for many of these parameters (see below). For the remaining system parameters that were not directly measured, we were able to derive numbers based on experimental data of steady-state concentrations and fluxes. A number of parameters were set such that the results of the model were in agreement with previous experimental data, specifically with the experimentally determined rate of β-catenin degradation (Salic et al. 2000; Lee et al. 2001). Finally, a few parameters had to be estimated; the constraint used was that the resulting model should be compatible with the steady-state and flux values. Table 1 lists the numeric values of all of the input quantities of the model. These quantities are either specific parameters, such as dissociation constants, or systemic properties, such as steady-state concentrations or fluxes, from which the other parameters have been derived. Both types of input quantities include experimental data as well as estimated values. The specific numerical values affect the model to differing degrees. In a later section, we analyze the effects of changing the values of the parameters around their reference numbers. The types of input data used for our modeling can be divided into five groups.

**Table 1:**
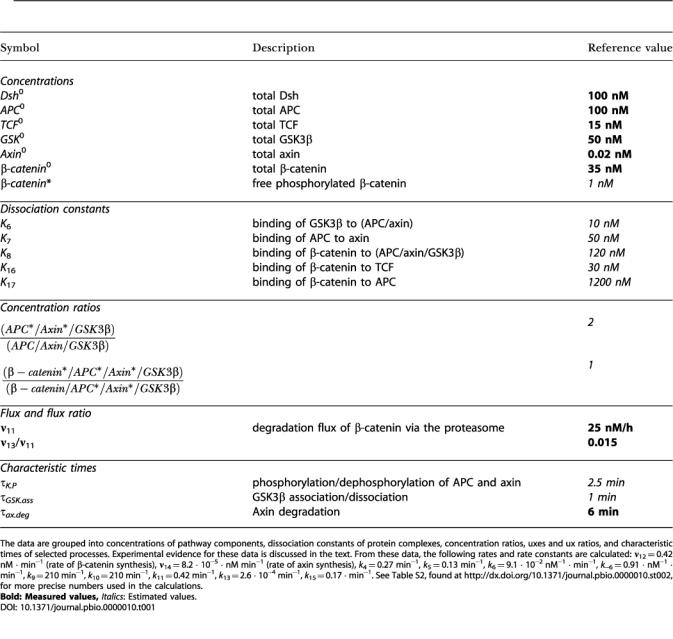
Numeric Values of Input Quantities of the Model for the Reference State

The data are grouped into concentrations of pathway components, dissociation constants of protein complexes, concentration ratios, fluxes and flux ratios, and characteristic times of selected processes. Experimental evidence for these data is discussed in the text. From these data, the following rates and rate constants are calculated: **ν**_12_ = 0.42 nM · min^−1^ (rate of β-catenin synthesis), **ν**_14_ = 8.2 · 10^−5^ · nM min^−1^ (rate of axin synthesis), *k*_4_ = 0.27 min^−1^, *k*_5_ = 0.13 min^−1^, *k*_6_ = 9.1 · 10^−2^ nM^−1^ · min^−1^, *k_−_*_6_ = 0.91 · nM^−1^ · min^−1^, *k*_9_ = 210 min^−1^, *k*_10_ = 210 min^−1^, *k*_11_ = 0.42 min^−1^, *k*_13_ = 2.6 · 10^−4^ min^−1^, *k*_15_ = 0.17 · min^−1^. See Table S2, found at http://dx.doi.org/10.1371/journal.pbio.0000010.st002, for more precise numbers used in the calculations

**Bold: Measured values,** *Italics*: Estimated values

The first group of input data contains both total concentrations (*Dsh* ^0^, *APC* ^0^, *TCF* ^0^, and *GSK3β* ^0^) and steady-state concentrations (*Axin* ^0^, *β-catenin* ^0^, β-catenin*). The total concentrations of Dsh, TCF, GSK3β, axin, β-catenin, and APC in Xenopus egg extract were determined experimentally using Western blot analysis by comparing the intensity of the signal to that of known amounts of recombinant protein. The concentration of phosphorylated β-catenin w as estimated because we have not been able to directly determine its level in extracts. However, we estimate that this value is small compared to that of nonphosphorylated β-catenin for the following reasons: (1) Addition of axin to Xenopus extracts dramatically increases the rate of β-catenin degradation. Since the role of axin is to promote phosphorylation of β-catenin, which is subsequently degraded, this would suggest that normally a significant pool of β-catenin exists in the nonphosphorylated form. (2) Western blot analysis of Xenopus extracts demonstrates that only a small percentage (<10%) of total β-catenin can be detected as migrating with a slower mobility, which likely represents the phosphorylated form of β-catenin.

The second group of input data was experimentally obtained from measurements of rates of dissociation of protein complexes. Binding constants were calculated based on the assumption that association rates approached that of the diffusion limits for a typical protein in an aqueous solution. The ratio *K*_17_/*K*_8_ = 10 of the dissociation constants characterizing the binding of β-catenin to APC and APC*/axin*/GSK3β, respectively, is based on previous experimental results demonstrating that β-catenin has a 10-fold lower affinity for nonphosphorylated compared to phosphorylated APC (Salic et al. 2000). In addition, we have shown experimentally (unpublished data; see Materials and Methods) that phosphorylated β-catenin dissociates from axin more rapidly (reaction 10) than nonphosphorylated β-catenin. Once phosphorylated, β-catenin will thus dissociate from the axin complex and undergo polyubiquitination and proteolysis.

The third group of input data consists of the two concentration ratios in the Destruction Core Cycle for complexes that either contain or lack β-catenin. The concentration ratio for the complexes that lack β-catenin is represented by the ratio of its phosphorylated versus nonphosphorylated forms and reflects the relative activities of its kinase(s) and phosphatase(s), respectively. By contrast, the concentration ratio of the two β-catenin-containing degradation complexes represents the relative activities of β-catenin phosphorylation and the rate of release of phosphorylated β-catenin from the complex. These parameters were chosen rather arbitrarily to indicate roughly equal kinase and phosphatase activities and yielded realistic values for the overall fluxes, given the known concentrations and kinetic rate constants.

The fourth group of data includes the steady state flux **ν**_11_ for the degradation of β-catenin via the Wnt pathway and the flux ratio **ν**_13_ */* **ν**_11_ describing the extent to which β-catenin is degraded via non-Wnt mechanisms (e.g., via Siah-1 and presenilin [Liu et al. 2001; Matsuzawa and Reed 2001; Kang et al. 2002]). We have now measured this Wnt pathway–independent degradation in Xenopus extracts (see Materials and Methods; value shown in Table 1).

The final group of input data consists of the characteristic time constant (τ) of selected processes. This is the time it takes for the concentration to drop to 1/e of its initial value. These characteristic times include τ*_K_* **_._** *_P_* = 1/(*k*_4_ + *k*_5_) for the kinase/phosphatase cycle that mediates phosphorylation/dephosphorylation of both APC and axin in the degradation complex (steps 4 and 5), τ*_GSK3β_* **_._** *_ass_* = 1 */* (*k*_6_ *GSK3β* + *k*_−6_) for the binding equilibrium of GSK3β with the APC/axin complex (step 6), and τ*_ax_* **_._** *_deg_* for axin degradation (step 15). Values for the rate of axin degradation were determined directly from experiments performed in Xenopus egg extracts (unpublished data). Experiments to determine the rate of APC and axin dephosphorylation (τ*_K_* **_._** *_P_* ≈ 2.5 min) were performed using in vitro ^32^P-labeled recombinant APC and axin. Radiolabeled proteins were added to Xenopus egg extracts, and the loss of radioactivity over time was assessed by SDS-PAGE and autoradiography (Salic et al. 2000). The legend to Table 1 contains the values of rate constants calculated from the input quantities using the described system of equations. The values of all variables in the reference state are listed in the first column of Table 2. These values represent the steady state solutions of system equations using the data in Table 1 as input quantities with the value of Wnt set at *Wnt* = 0.

**Table 2:**
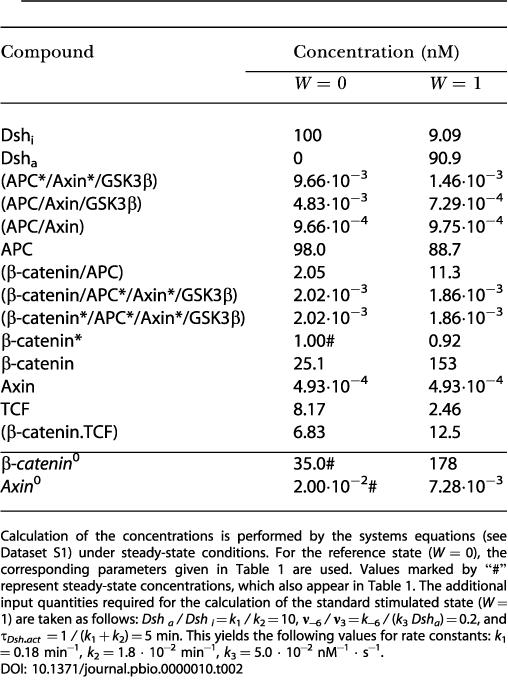
Steady-State Concentrations of Pathway Compounds in the Reference State and in the Standard Stimulated State

Calculation of the concentrations is performed by the systems equations (see Dataset S1) under steady-state conditions. For the reference state (*W* = 0), the corresponding parameters given in Table 1 are used. Values marked by “#” represent steady-state concentrations, which also appear in Table 1. The additional input quantities required for the calculation of the standard stimulated state (*W =* 1) are taken as follows: *Dsh _a_ / Dsh _i_ = k*_1_ */ k*_2 _= 10, **ν**_−6_ */* **ν**_3_ *= k*_−6_ */* (*k*_3_ *Dsh_a_*) = 0.2, and τ*_Dsh_* **_._** *_act _* = 1 */* (*k*_1_ *+ k*_2_) = 5 min. This yields the following values for rate constants: *k*_1_ = 0.18 min^−1^, *k*_2_ = 1.8 · 10^−2^ min^−1^, *k*_3_ = 5.0 · 10^−2^ nM^−1^ · s^−1^

Using the reference state as a starting point, we consider other stationary states that are attained when the pathway is permanently stimulated. To describe the strength of Wnt stimulation, we introduce a dimensionless quantity *W = Wnt/Wnt* ^0^ that represents the ratio of the Wnt concentration with respect to its concentration *Wnt* ^0^ in a “standard” stimulated (signaling) state. *W =* 0 and *W =* 1 characterize the reference state and a standard stimulated state, respectively, with the hyperstimulated state defined as *W* > 1. In order to calculate concentrations in the standard stimulated state, additional input quantities are required. These include the ratio of the active and inactive forms of Dsh (*Dsh_a_/Dsh_i_*), the relation between non-Dsh-mediated and Dsh-mediated release of GSK3β from the destruction complex (the flux ratio **ν** *_−_*_6_/**ν**_3_), and the characteristic time for the Dsh activation/inactivation cycle (τ*_Dsh_* **_._** *_act_*). These values are not at present measurable. The values for these input quantities are listed in the legend of Table 2. In a later section, we analyze the effects of changes in these additional input quantities.

By setting *W* = 1 and fixing all other parameters, we arrive at steady-state solutions of the systems equations (see Dataset S1, Equations. [A-1]–[A-15]), which yield the numerical variables for the standard stimulated state (listed in the second column of Table 2). A comparison of this state with the reference state shows that the concentration of free nonphosphorylated β-catenin increases by a factor of approximately 6, from 25 to 153 nM. Upon Wnt stimulation, the free phosphorylated β-catenin concentration decreases by 8%, from 1 nM to 0.92 nM. The increase in β-catenin levels reflects the decrease in its degradation caused by the reduction in the ability of GSK3β to phosphorylate it. The concentration of the β-catenin/TCF complex increases by a factor of 1.8. The large increase in β-catenin concentration shifts the binding equilibrium between APC and β-catenin and the concentration of free APC falls slightly. Total axin concentration decreased by a factor of 2.7 upon Wnt stimulation since addition of Dsh decreases the concentration of the various axin containing complexes. Remarkably, the steady-state concentration of free axin is constant during the transition from *W* = 0 to *W* = 1. This is due to the fact that under steady-state conditions, the rate of axin synthesis equals its degradation; the rate of synthesis (**ν**_14_) is a fixed value and the rate of degradation depends solely on the concentration of free axin (and independent of other parameters such as binding constants and strength of the Wnt signal).

As expected, simulations of increasing Wnt activation (0 ≤ *W* ≤ 1.4) on the steady-state concentrations of β-catenin and axin reveal a nearly hyperbolic saturation of increasing concentrations of nonphosphorylated and total β-catenin with increasing strength of Wnt stimulation. Furthermore, Wnt stimulation affects the steady-state concentrations of axin and β-catenin in an opposite direction (see Figure S1).

### β-Catenin Degradation: Comparison of Theory and Experiment

**Figure 2:**
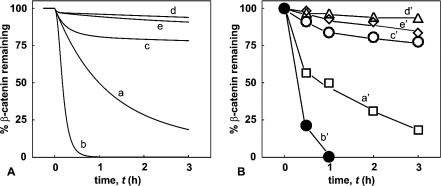
Kinetics of β-Catenin Degradation: Simulation and Experimental Results (A) Simulated timecourses of β-catenin degradation. The straight line for *t <* 0 corresponds to the reference state of β-catenin using the parameters given in the legends of Table 1 and 2. In vitro conditions are simulated by switching off synthesis of β-catenin and axin (**ν**_12_ = 0, **ν**_14_ = 0 for *t* ≥ 0). Curve a: reference case (no addition of further compounds); curve b: addition of 0.2 nM axin; curve c: addition of 1 μM activated Dsh (deactivation of Dsh was neglected, *k*_2_ = 0); curve d: inhibition of GSK3β (simulated by setting *k*_4_ = 0, *k*_9_ = 0); curve e: addition of 1μM TCF. Addition of compounds (axin, Dsh, TCF) and inhibition of GSK3β was performed at *t* = 0. (B) Experimental timecourse of β-catenin degradation in Xenopus egg extracts in the presence of buffer (curve a′), axin (curve b′: 10 nM), Dsh (curve c′: 1 μM), Li^+^ (curve d′: 25 mM), or Tcf3 (curve e′: 1 μM).

To test whether the mathematical model represented the Wnt pathway under a variety of conditions, we ran through a series of simulations, all of which used the same set of parameters. From these we calculated simulated timecourses for β-catenin degradation under a range of different conditions (increased axin concentration, increased Dsh^a^ concentration, inhibition of GSK3β, increased TCF concentration) (Figure 2A). We then tested the results using the previously described biochemical system (Salic et al. 2000; Lee et al. 2001), adding purified proteins or compounds at *t =* 0 (Figure 2B). Simulations and experimental results are each shown as plots of total β-catenin concentration versus time. The agreement between theory and experiment is excellent.

The straight line for *t <* 0 represents the reference state. The simulated reference state curve (Figure 2A, curve a) for β-catenin degradation is calculated for *t >* 0, at which there is an absence of protein synthesis for axin (**ν**_14_ = 0) and β-catenin (**ν**_12_ = 0). This reference curve is in close agreement with our experimental data (Figure 2B, curve a′) with identical half-lives for β-catenin degradation (theoretical value of *t*_½_ = 60.2 min versus experimental value of *t*_½_ = 60 min). We examined a new state, where we have increased the amount of endogenous axin (0.02 nM) by 0.2 nM. As shown in Figure 2A, curve b, the additional axin markedly accelerated β-catenin degradation (*t*_½_ = 11.8 min) in agreement with the experimentally obtained values (Figure 2B, curve b′; *t*_½_ = 12 min). Theoretically, the effect of axin on β-catenin degradation is primarily due to the large concentration difference between the two scaffold proteins, APC and axin. Owing to the high concentration of APC, an increase in axin concentration results in a sharp increase in the concentration of the APC/axin complex, thereby accelerating β-catenin binding to the destruction complex.

Curve d in Figure 2 shows the effect of inhibiting GSK3β on β-catenin degradation. This effect is produced in the simulation by inhibiting GSK3β activity (steps 4 and 9). Only a small fraction of β-catenin (phosphorylated β-catenin) is available for degradation after complete inhibition of β-catenin phosphorylation (step 9), so inhibition is rapid. This is in complete agreement with our experimental data in which degradation is essentially blocked after inhibiting GSK3β activity by lithium (Figure 2B, curve d′). Curve e in Figure 2A predicts that β-catenin degradation is strongly inhibited after the addition of 1 μM TCF. Previously we have shown that β-catenin is sequestered by TCF, thereby resulting in a significant decrease in free β-catenin (Lee et al. 2001). The addition of TCF would be expected to decrease the rate of β-catenin phosphorylation (step 9) and subsequently β-catenin degradation. This is also seen experimentally (Figure 2B, curve e′).

The immediate inhibition by LiCl is in contrast with the action of Dsh that inhibits only after a significant delay. We were intrigued by the theoretical biphasic degradation curves of β-catenin in the presence of Dsh_a_, as well as the experimental support for it (Figure 2A and 2B, curves c and c′). In both cases, there is an initial rapid decrease in β-catenin in the first 30 min to 1 h, followed by a much slower decrease. Such a feature should allow us to distinguish mechanistic details of complex formation. Experimentally, the biphasic nature of Dsh activity is not due to a delay in Dsh activation upon its addition to the Xenopus extracts since we see the same effect with Dsh protein that has been “activated” with extracts prior to its use in our degradation assay. As shown in Table 1, the characteristic time τ*_K_* **_._** *_P_* of phosphorylation and dephosphorylation of APC and axin in the destruction complex is relatively slow (2.5 min), and it therefore takes 5 min for 75% of the complex to be dephosphorylated. If Dsh_a_ acted only on the dephosphorylated complex (through step 3) to remove GSK3β and thus block phosphorylation of the complex, then we would predict the biphasic kinetics shown in Figure 2A, curve c. These data suggest that Dsh inhibits the phosphorylation of the scaffold complex by GSK3β, but does not inhibit the phosphorylation of β-catenin. When Dsh binds, the complex can go around many times binding and phosphorylating β-catenin before it dissociates and is inhibited by Dsh. One hour after the addition of Dsh, β-catenin degradation is significantly inhibited due to the removal of a significant pool of GSK3β from the degradation complex over time (through the action of Dsh). As a result, the scaffold protein axin is dephosphorylated by the phosphatase (step 5) that remains bound to the degradation complex. Dephosphorylated axin is rapidly ubiquitinated and degraded when the β-catenin degradation normally stops. The small decrease in β-catenin levels in Figure 2, curve c, after a 1 h incubation with Dsh, is due to degradation of β-catenin via nonWnt pathway mechanisms (see Table 1) that we have incorporated into our model.

**Figure 3:**
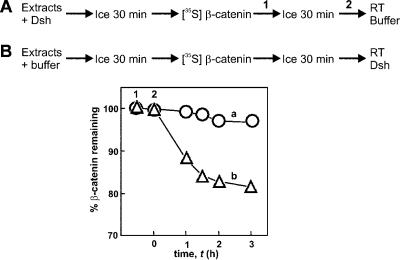
Preincubation of Dsh in Xenopus Egg Extracts Abolishes the Lag in Dsh Activity Labeled β-catenin was incubated in Xenopus extracts on ice 30 min prior to (B) or 30 min after (A) the extract had been preincubated with 1 μM Dsh. No degradation of the labeled β-catenin was detected while the reactions were on ice. The reactions were started by shifting to 20°C.

To test this prediction beyond consistency with experimental data, we performed an experiment in which Dsh was either preincubated with extract before or added at the same time as radiolabeled β-catenin (Figure 3). If β-catenin and Dsh are added at the same time, there is an initial rapid loss of β-catenin (Figure 3, curve b) followed by pronounced inhibition of degradation after 1 h. This initial rapid loss is consistent with Dsh acting on a subpopulation of degradation complexes (presumably the unphosphorylated forms). Strikingly, preincubation with Dsh prior to the addition of radiolabeled β-catenin (Figure 3, curve a) results in immediate action of Dsh. We interpret this result to simply reflect the fact that over time in the preincubated extract Dsh can remove GSK3β from the degradation complexes, thereby enhancing the activity of the phosphatase and, as a result, promoting the degradation of axin and inhibition of β-catenin degradation. The small decrease in β-catenin levels at *t* > 2 h in both curves a and b again suggests the existence of a slow degradation process mediated by non-Wnt pathway mechanisms.

### Clues to Axin Activity from Its Very Low Cellular Concentration

**Figure 4:**
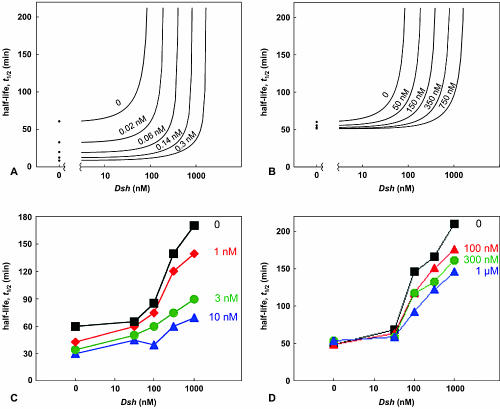
The Effect of Dsh versus Axin or GSK3β on the Half-Life of β-Catenin in Xenopus Extracts (A and B) Predicted effects of Dsh, axin, and GSK3β on the half-life of β-catenin degradation. The half-lives are calculated from simulated degradation curves. Data are plotted as function of added Dsh (logarithmic scale) for various concentrations of axin (A) and GSK3β (B). (C and D) Measured effects of Dsh, axin, and GSK3β on the half-life of β-catenin degradation. Stimulation of β-catenin degradation by axin occurs throughout the range of Dsh concentrations tested. (C) Axin increases the rate of β-catenin degradation even in the absence of added Dsh. (D) Stimulation of β-catenin degradation by GSK3β is detected only at high concentrations of Dsh. No effects of GSK3β on β-catenin degradation can be detected at less than 30 nM added Dsh. There is a disparity between the concentrations of axin in the experimental and theoretical curves. We assume that this is most likely due to the specific activity of the expressed axin protein.

In establishing quantities for our model in Table 1, we found that the axin concentration (20 pM) is much lower than the concentration of the other major components (β-catenin, 35 nM; APC, 100 nM; Dsh, 100 nM; and GSK3β, 50 nM). This unusual finding suggests that the function of the Wnt signaling system may actually depend on a low axin concentration. Our theoretical predictions for the effects of axin, GSK3β, and Dsh on the half-lives of β-catenin are shown in Figure 4A and 4B, respectively. At zero concentration of Dsh, doubling the concentration of axin (from the reference state, indicated as 0, to a state where the concentration has been increased by 0.02 nM) causes a 50% drop in the half-life of β-catenin. By contrast, a doubling of the GSK3β concentration only decreases the half-life of β-catenin by 10%. The small effect of GSK3β is predicted to be due to the fact that only a limited amount of axin can be recruited to the degradation complex through binding to additional GSK3β. On the other hand, increased axin concentrations are immediately translated into an increased concentration of the destruction complex, because the concentrations of APC and GSK3β are high. Changing the concentration of either GSK3β or of axin should also change the amount of Dsh_a_ required to inhibit β-catenin degradation, but the pathway is much more sensitive to axin concentration than it is to GSK3β concentration. In the presence of high concentrations of axin, the effect of Dsh_a_ should be blocked; high concentrations of axin will lead to high concentrations of the phosphorylated destruction complex no matter what level of Dsh_a_ activity is present. High levels of the destruction complex will require even higher levels of Dsh to overcome the inhibition. The interaction between Dsh_a_ and GSK3β is similar in principle: Dsh-mediated release of GSK3β (step 3) from the degradation complex can simply be reversed by sufficiently high concentrations of GSK3β (step 6). In this case, however, the effect is small. Thus, axin blocks the action of Dsh so effectively that it renders the Dsh pathway inoperable.

In Figure 4C and 4D, we studied experimentally the dose-dependent effects of Dsh, GSK3β, and axin on β-catenin degradation. These curves represent β-catenin half-lives for various concentrations of axin (Figure 4C) and GSK3β (Figure 4D) with varying concentrations of Dsh. The results are qualitatively similar to those predicted by the model. As expected, β-catenin degradation is inhibited by increasing Dsh concentration and stimulated by increasing the concentration of either axin or GSK3β. There are, however, two pronounced differences in the effects of axin and GSK3β on Dsh inhibition. Whereas axin activates β-catenin degradation over a wide range of Dsh concentrations (Figure 4C), the effect of GSK3β becomes significant only at high concentrations of Dsh (Figure 4D). Furthermore, the inhibitory effect of Dsh can be almost completely blocked by high concentrations of axin (10 nM). In contrast, GSK3β (1 μM) can only partially inhibit the strong inhibitory effect of Dsh on β-catenin degradation.

Our experimental results, however, show a smaller effect on the half-life of β-catenin degradation at high concentrations of Dsh as GSK3β levels are increased. Also, the concentrations of added axin in the theoretical curve and the experimental curves are very different. The quantitative difference between the model and experimental may simply reflect the fact that the specific activity of our GSK3β and axin preparations (purified from *Sf9* cells and bacteria, respectively) may be low and that a significant fraction of the recombinant proteins may not be active. Alternatively, the low activity of GSK3β may point to an unidentified inhibitory activity present in our Xenopus egg extracts.

### Effects of Dynamic Changes in Protein Concentrations

The dependence of flux on the concentration of a pathway component is a measure of how much the flux is sensitively controlled by that component. In metabolic control theory, the normalized concentration-dependent parameters of the total flux known as control coefficients have been very useful in defining the characteristics of pathways (Heinrich and Rapoport 1974; Fell 1997). Similarly, in the analysis of bacterial chemotaxis, the response of a behavioral parameter as a function of changes in specific kinetic rates has been termed robustness (Alon et al. 1999). Such terms are rarely measured in signal transduction.

To determine the effects of changes in the levels of Wnt pathway components, we analyzed how the flux (β-catenin degradation) changes with changes in the concentrations of *APC* ^0^, *GSK3β* ^0^, *Dsh* ^0^, and *TCF* ^0^ (see Figure S2).

We chose to focus on the effects of changes in the concentrations of pathway components in the reference state, because similar effects were also seen for the stimulated state. Recently, we investigated a new and important property of the Wnt pathway, namely that the degradation of axin (reaction 15) is dependent on APC (unpublished data). The degradation rate of axin is mathematically expressed in the following manner:

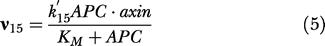
where *K_M_* represents a half-saturation constant for the activating effect of APC.

**Figure 5:**
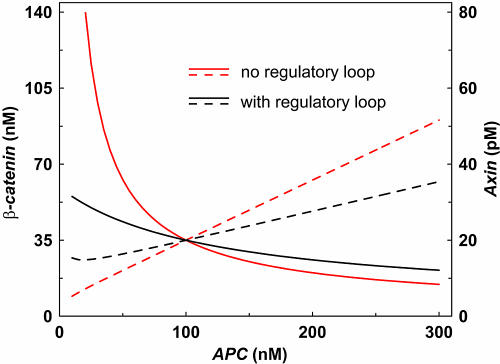
Effect of the Regulatory Loop for Axin Degradation The case “with regulatory loop” takes into account that axin degradation is APC-dependent (black curves). Alternatively, the case without this regulatory loop is considered (red curves). For the regulatory loop, the rate law (5) is used assuming that in the reference state the APC activation is half of its maximum (*K_M_* = 98.0 nM). The value of *k*′_15_ was chosen such that in the reference state both cases, with and without regulatory loop, yield the same degradation rate of axin (*k*′_15_ = 0.33 min^−1^).

The theoretical effect of APC on the concentrations of both β-catenin and axin is shown in Figure 5, where we considered independently the effect of APC-mediated degradation of axin (“with regulatory loop” where Equation [5] is applied) or the absence of such an effect (where the linear rate equation **ν**_15_ *= k*_15_ *axin* is applied). With APC-mediated axin degradation, β-catenin degradation is affected very little by changes in the concentration of APC (25% decrease with a 2-fold increase in APC concentration). This resistance to changes of β-catenin levels upon changes in APC concentration is due to the APC-dependence of axin degradation (see Figure 1 and Equation [5]). Decreasing the concentration of APC inhibits the degradation of axin, thereby promoting the formation of the degradation complex. As shown in Figure 5, in the absence of the regulatory loop, axin degradation is APC independent, homeostasis is lost, and β-catenin levels are greatly upregulated with decreasing APC concentrations. A comparison of the curves that represent the dependence and independence of axin degradation on APC (dashed lines in Figure 5) indicates that the regulatory loop acts in such a way that the normally inhibitory effect on β-catenin degradation as a result of lowering the concentration of APC is counteracted by an increase in axin levels.

We have also simulated the effects of changes in the rate of β-catenin (**ν**_12_) and axin (**ν**_14_) synthesis on both β-catenin and axin levels (see Figure S3). Interestingly, changing the level of axin or β-catenin affects the concentration of the other component in different ways. An increase in the synthesis of axin results in a decrease in β-catenin, whereas increasing β-catenin synthesis leads to an increase in axin levels. This latter effect contrasts with effects observed upon changes of other parameters (see Figure S2) that affect the concentrations of axin and β-catenin in opposite directions.

### Transient Stimulation of the Pathway

Wnt stimulation in vivo is transient, likely due to receptor inactivation/internalization and/or other downregulatory processes.

We model transient Wnt stimulation by an exponential decay:


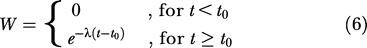


where the reciprocal of *λ* represents the characteristic lifetime τ*_W_* of receptor stimulation and *t*_0_ denotes the onset of signaling. The concentration changes of all other pathway compounds resulting from Wnt stimulation can be calculated by numerical solution of the system equations (see Dataset S1), with initial values of the variables corresponding to the reference state.

**Figure 6:**
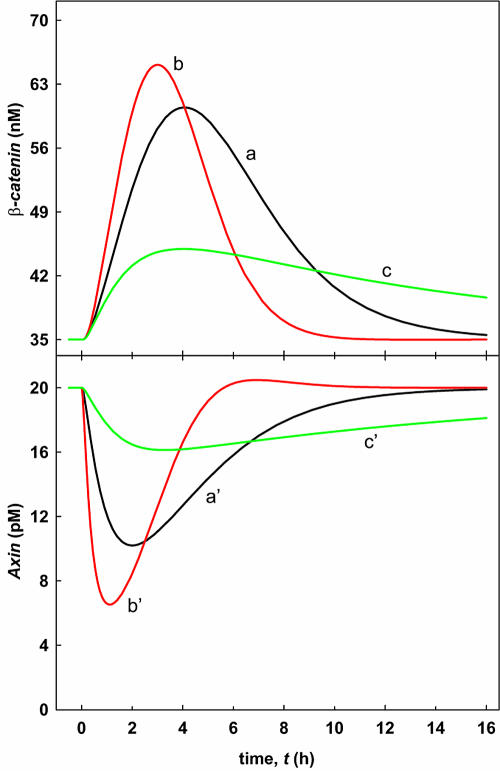
Timecourse of β-Catenin and Axin Concentrations Following a Transient Wnt Stimulation Transient activation of the pathway is modeled assuming a Wnt stimulus that decays exponentially (Equation [6] with τ*_W_* = 1/*λ* = 20 min) starting at *t*_0_ = 0. The straight line for *t* < 0 corresponds to the steady state before pathway stimulation. The curves are obtained by numerical integration of the differential equation system (see Dataset S1). The various curves for β-catenin and for axin differ in the turnover rate of axin determined by the parameters **ν**_14 _and *k*_15_ (curves a: reference values of these parameters; curves b: increase by a factor of 5; curves c: reduction by a factor of 5). All other parameters are given in the legend of Table 2.

Regulating axin turnover is important for Wnt signaling. Wnt-stimulated axin turnover has been reported in cultured mammalian cells (Yamamoto et al. 1999) and in *Drosophila* (Tolwinski et al. 2003). In a future paper we will show that axin turnover is affected inversely to β-catenin turnover by phosphorylation by GSK3β. Here we show theoretically that this regulated axin turnover sharply affects the dynamics of the response. Figure 6 shows the time-dependent behavior of the total concentration of β-catenin and the total concentration of axin upon transient Wnt stimulation. The concentration of β-catenin increases transiently and then returns to its initial value. In contrast, the concentration of axin is temporarily downregulated. Further analysis of Figure 6 reveals that the amplitude of the β-catenin signal upon transient stimulation is significantly lower than the steady-state concentration upon permanent stimulation (*W =* 1; see Figure S1). The curves a and a′ in Figure 6 are calculated for the reference values of the rate of axin synthesis and of the rate constant of axin degradation, whereas the curves b and b′ and the curves c and c′ are obtained for the case where both parameters are increased by a factor of 5 and decreased by a factor of 5, respectively. Under these conditions, both the degradation rate and the synthesis rate are altered by the same factor, thus maintaining essentially identical steady-state concentrations of axin. As a result, the steady-state concentrations of axin are the same in the unstimulated condition (*W =* 0) and after diminution of the Wnt signal; however, during active signaling, the differences in the dynamic nature of signal output at differing rates of axin turnover are dramatically revealed.

Interestingly, an increase in the turnover rate of axin leads to higher amplitudes and shorter durations of the β-catenin signal. This can be explained by the faster degradation of axin after its Dsh-mediated release from the destruction complex.****Thus, β-catenin degradation is effectively inhibited for a certain time period due to a reduced availability of the scaffold axin.

Since the steady-state concentration of free axin remains unchanged (rate of axin synthesis equals the rate of its degradation) during the transition from *W =* 0 to *W =* 1, a fast axin turnover favors rapid replenishment of the axin pool after the decline of the Wnt stimulus and, in this way, fast recovery of the destruction complex. This explains why the β-catenin signal is not only amplified, but becomes more spike-like. Increasing the turnover rate of axin affects the response of axin to temporary Wnt stimulation in a similar way as the response of β-catenin; i.e., the signal is amplified and sharpened (Figure 6). Closer inspection of Figure 6 reveals that the axin response precedes the β-catenin response. For example, in the reference case, the β-catenin concentration reaches its maximum at about 260 min (curve a), whereas the minimum of the axin concentration is reached at 130 min (curve a′). This effect can be understood by observing that it is the lowering of the axin concentration that decreases the concentration of the destruction core complexes; in turn, this stabilizes β-catenin.

### Mechanistic Differences between APC and Axin as Scaffolds

**Figure 7:**
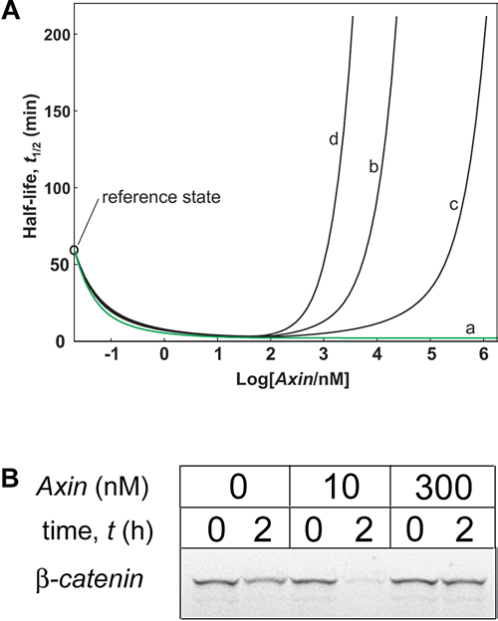
Effects of Increasing Axin Concentration on β-Catenin Degradation (A) Effect of axin concentration on β-catenin half-life. Curve a: reference case (*K*_18_, *K*_19_ > 1 nM, ordered mechanism); curve b: *K*_18_ = 1 nM, *K*_19_ > 1 nM; curve c: *K*_18_ > 1 nM, *K*_19_ = 1 nM; curve d: *K*_18_ = 1 nM. (B) High concentration of axin inhibits β-catenin degradation in Xenopus egg extracts. Labeled β-catenin was incubated in Xenopus extracts in the absence (0 nM) or presence of moderate (10 nM) and high (300 nM) concentrations of axin. Moderate concentrations of axin greatly accelerate, whereas high concentrations inhibit β-catenin degradation.

As the axin concentration is several orders of magnitude lower than that of the other components in the degradation pathway (see Table 1), we decided to test the effect of increasing axin levels (up to, equal to, and greater than the concentrations of other components in the pathway). To do this, we had to extend the model to include additional reactions, marked in blue in Figure 1; these had previously been neglected due to the very low axin concentrations. High axin concentrations affect most prominently the formation of the β-catenin/axin complex. Assuming a realistic value for the β-catenin–axin dissociation constant (*K*_18_ *=* 1 nM), a moderate increase in axin concentration should theoretically accelerate β-catenin degradation, whereas a much higher concentration should result in inhibition of β-catenin degradation, due to the formation of partial complexes on axin. A more extensive analysis of β-catenin half-lives over a range of axin concentrations shows such a biphasic curve (Figure 7A, curve b). These effects can also be seen experimentally in extracts (Figure 7B), where 10 nM axin accelerates and 300 nM axin inhibits β-catenin degradation. The *t*_½ _decrease for low amounts of added axin can be easily explained by the fact that greater amounts of APC and GSK3β can be recruited to form the destruction complex. As a result, the *t*_½_ decreases from 60 min to *t*_½_ = 3 − 4 min. The inhibitory effect of axin becomes apparent only for axin concentrations approaching that of the other components. As shown in Figure 7A, the effect of axin binding only to GSK3β (*K*_19_ = 1 nM, *K*_18_ → ∞) only becomes inhibitory at higher than micromolar concentration (curve c), whereas the combined effect of binding to both β-catenin and GSK3β (*K*_18_ = 1 nM, *K*_19_ = 1 nM) shows inhibition at less than 500 nM (curve d). If, however, we model an ordered process of binding to axin, then abortive inhibitory complexes cannot form. We show this in Figure 7A. Here there is no separate binding of axin to β-catenin or GSK3β. In this case, there is no increase in the t_½_ at high axin concentrations (Figure 7A, curve a).

**Figure 8:**
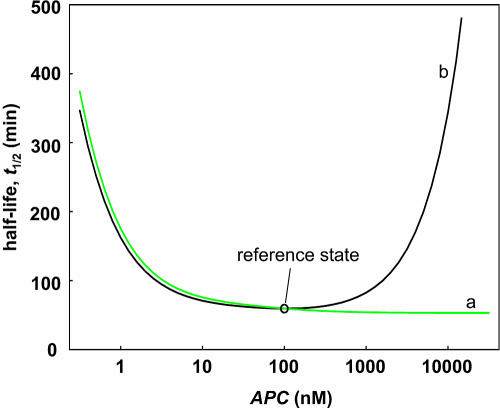
Effects of APC Concentrations on β-Catenin Degradation Effect of APC concentration on β-catenin half-life assuming an ordered (curve a) or nonordered mechanism (curve b: *K*_17_ = 1,200 nM), respectively.

We also examined theoretically the effects of increasing APC concentration on the half-life of β-catenin, as shown in Figure 8. The black curve corresponds to a nonordered mechanism, such as that found in axin, in which the β-catenin–APC dissociation constant (reaction 17) is low. The inhibitory effect of APC at high concentrations is due to its β-catenin buffering activity. The green curve corresponds to an ordered mechanism and reflects a high β-catenin–APC dissociation constant (high *K*_17_). In this case, increasing concentrations of APC greater than the reference concentrations does not lead to inhibition of β-catenin degradation even at very high concentrations of APC. In cultured cells, overexpression of APC stimulates β-catenin degradation (Munemitsu et al. 1995; Papkoff et al. 1996). Unfortunately, we are presently unable to express full-length APC in Xenopus egg extracts to measure the effects of high levels in the extract system.

**Figure 9:**
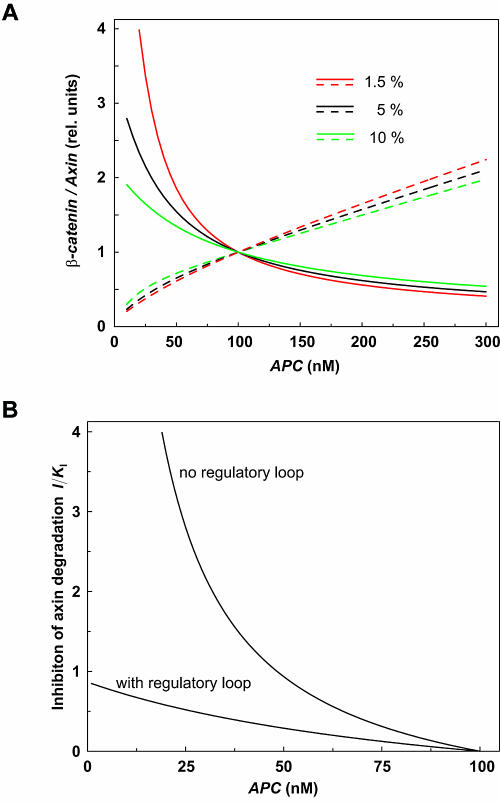
Effects of the Alternative β-Catenin Degradation Pathway and of Axin Degradation at Low Concentrations of APC (A) The alternative β-catenin degradation pathway (axin independent) can have profound effects on β-catenin levels at low APC concentrations. Variations of β-catenin and axin resulting from changes in APC concentration were calculated from the standard stimulated state. Relative variations were plotted since variation in the share of alternative degradation (1%, 5%, and 10%) results in changes of the standard stimulated state (all parameters are constant). β-Catenin and axin levels for varying contributions of the alternative degradation pathway are as follows: 1.5%, β-catenin 178 nM, axin 0.00728 nM; 5%, β-catenin 151 nM, axin 0.00679 nM; 10%, β-catenin 125 nM, axin 0.00629 nM. (B)**** Inhibition of axin degradation reduces β-catenin concentration after loss of APC. Plotted is the concentration of a potential proteasome inhibitor I (scaled to its inhibition constant, *K*_I_) necessary to reduce β-catenin concentration to its original level, depending on the concentration of APC.

β-Catenin can also be degraded by nonaxin-dependent mechanisms, which include Siah-1 and presenilin-mediated degradation. Though they are expected to contribute very little to the total flux through the pathway, the nonaxin-dependent processes may have very important influences under certain conditions. In our Xenopus system, these alternative pathways do not contribute greatly to the half-life of β-catenin. Experimentally, we have measured only a 1.5% contribution to total β-catenin degradation such that the half-life of β-catenin is 45 h when the axin-dependent processes are inhibited. If in some situations the nonaxin-dependent degradation contributed 10% to the flux, the half-life would be 6.3 h (*k*_13_ = 1.83 · 10^−3^ min^−1^). The alternative pathways have very little effect on the half-life of β-catenin at normal and supranormal concentrations of APC. However, the effect of these alternative pathways becomes much more prominent when the APC concentration is lowered, a situation that may be significant under pathological conditions. As seen in Figure 9A, when APC levels are at 50% of their normal concentration, there are dramatic differences in β-catenin concentration, depending on whether the alternative degradation pathway contributes to 1.5% or 10% of the total β-catenin degradation activity. The importance of the regulatory loop involving APC-mediated axin degradation is shown in Figure 9B. In the absence of the regulatory loop, a significant inhibition of APC levels would strongly inhibit axin degradation, leading to a large increase in β-catenin levels. The control of β-catenin would be very brittle in this circumstance. However, by making axin degradation dependent on APC, a loss of APC would not stabilize axin levels, and the high axin levels would support continued degradation of β-catenin. This is the situation labeled “with regulatory loop” shown in Figure 9B. The control of axin degradation could be a decisive factor in the response of the system to genetic or environmental effects on APC.

### Control, Modular Composition, and Robustness of the Wnt Pathway

The model contains many parameters that affect the system behavior in different ways and to various extents. We can systematically investigate these parameters and look for those whose perturbation the system is most sensitive or most robust against. We focus on the concentrations of β-catenin and axin and calculate the responses in the total concentrations of these two compounds upon changes in the rates of the individual processes. For quantifying the effects of the rate constants *k_+i_* and *k_−i_*, we use control coefficients for the total concentration of β-catenin


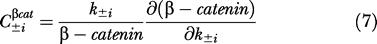


and corresponding definitions for the control coefficients *C^axin^_±i_* for the total axin concentration. These coefficients, originally proposed for quantifying control in metabolic networks (for reviews, see Heinrich and Schuster 1996; Fell 1997), describe the relative changes of the concentrations of the given compounds to relative changes of the rate constants. The control coefficients for the reference state are listed in Table 3. It should be remembered that the following discussion refers to small perturbations of the reference state.

**Table 3:**
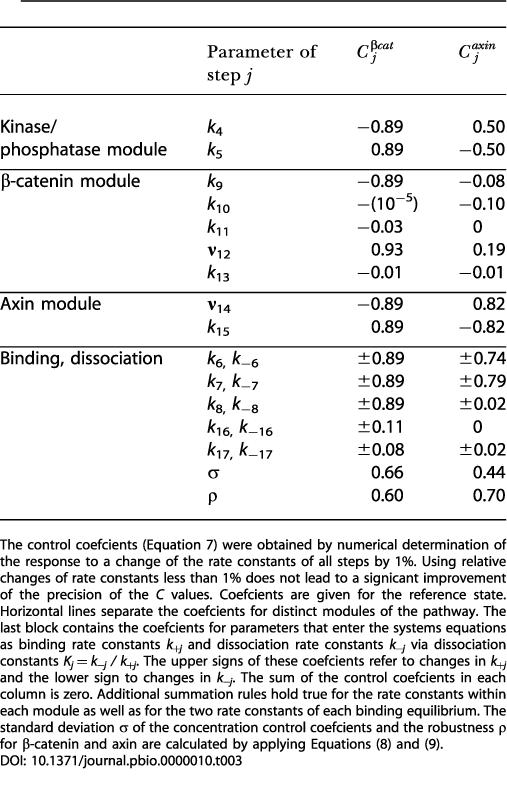
Control Coefficients for the Total Concentrations of β-Catenin and Axin and Parameters Quantifying the Sensitivity and the Robustness of the Wnt/β-Catenin Pathway

The control coefficients (Equation 7) were obtained by numerical determination of the response to a change of the rate constants of all steps by 1%. Using relative changes of rate constants less than 1% does not lead to a significant improvement of the precision of the *C* values. Coefficients are given for the reference state. Horizontal lines separate the coefficients for distinct modules of the pathway. The last block contains the coefficients for parameters that enter the systems equations as binding rate constants *k_+j_* and dissociation rate constants *k_−j _*via dissociation constants *K_j_* = *k_−j_ / k_+j_*. The upper signs of these coefficients refer to changes in *k_+j_* and the lower sign to changes in *k_−j_*. The sum of the control coefficients in each column is zero. Additional summation rules hold true for the rate constants within each module as well as for the two rate constants of each binding equilibrium. The standard deviation *σ* of the concentration control coefficients and the robustness *ρ* for β-catenin and axin are calculated by applying Equations (8) and (9)

For the reference state, there are six steps exerting strong negative control on the total β-catenin concentration (*C^βcat^_i_* ≅ −1). This group includes the reactions participating in assembling the destruction complex APC*/axin*/GSK3β. The corresponding parameters involve the rate constants *k*_7_ for the binding of axin to APC, *k*_6_ for the association of GSK3β to the APC/axin complex, and *k*_4_ for the phosphosphorylation of axin and APC in the destruction complex. Similar strong negative control is exerted by β-catenin binding to the phosphorylated destruction complex (rate constant: *k*_8_), the phosphorylation of β-catenin in the destruction complex (rate constant: *k*_9_), and the synthesis of axin (**ν**_14_).

Six other reactions exert strong positive control in the reference state on the total concentration of β-catenin (concentration (*C^βcat^_i_* ≅ 1). To this group belong the reactions participating in the disassembly of the destruction complex APC*/axin*/GSK3β, which are described by the rate constants *k_−_*_7_ for the dissociation of the APC/axin complex, *k_−_*_6_ for the dissociation of GSK3β from the destruction complex, and *k*_5_ for the dephosphorylation of the APC and axin in the destruction complex. Other steps with a high positive control are the dissociation of β-catenin from the destruction complex (rate constant: *k_−_*_8_), axin degradation (rate constant: *k*_15_), and β-catenin synthesis (**ν**_12_).

There are many reactions exerting almost no control on β-catenin levels in the reference state. This group includes binding of β-catenin to TCF and APC (*k*_16_ and *k*_17_), and the corresponding dissociation processes (*k_−_*_16_ and *k_−_*_17_; again only valid for small perturbations). Interestingly, the effects of the two β-catenin degradation processes (rate constants: *k*_11_ and *k*_13_) are also small. Calculation of control coefficients for the standard stimulated state reveals that some steps that exert no control in the reference state become important. These are the activation and inactivation of Dsh (rate constants: *k*_1_ and *k*_2_) and, more pronounced, the Dsh-mediated release of GSK3β from the destruction complex (*k*_3_). For all other processes, the signs of the control coefficients for β-catenin and axin do not change at the transition from the reference state to the standard stimulated state. The effects of parameter changes on axin are generally opposite to those on β-catenin; i.e., processes with a positive control coefficient for β-catenin have negative control coefficients for axin and vice versa. A significant exception is the synthesis of β-catenin, which exerts a positive control not only on β-catenin but also on axin, as expected from the results obtained in the last section.

Closer inspection of Table 3 reveals that the values of the control coefficients for the rate constants sum up to zero. This fact is known as the summation theorem for concentration control (Heinrich and Rapoport 1974) and is valid for all reaction networks at steady state. This result finds its explanation in the invariance of the steady-state concentrations against simultaneous change of all rate constants by the same factor. Interestingly, in the present case there are subgroups of processes whose control coefficients separately sum up to zero, indicating a modular structure of the pathway. In Table 3, the control coefficients of the different modules are separated by horizontal lines. The main four subgroups are the Dsh module (not shown in Table 3), the kinase/phosphatase module, the β-catenin module, and the axin module. A subgroup is defined by a set of reactions where the control coefficients of the binding reactions are opposite to those of the corresponding dissociation reactions (*C_+i_* = −*C_−i_* for *i* = 6, 7, 8, 16, 17).

For those more familiar with genetic manipulation, it is more common to vary the concentrations of individual components rather than vary the rate constant of a reaction. Table 4 shows the control coefficients for β-catenin and axin calculated for changes in the total concentrations of pathway components instead of the rate constants. Using the values of Table 4, the potential tumor-supressing effects (of APC, GSK3β, and axin) and potential oncogenic effects (of PP2A, TCF, Dsh, β-catenin) can be explained and quantified. It may be worth mentioning that there is no summation theorem for the control coefficients when calculated by changing total concentrations instead of rate constants. For practical reasons, it may be easier to discuss the coefficients with respect to concentration changes (Table 4); for theoretical reasons, changing rate constants are simpler to handle. We think that eventually it will also be clearest to speak about oncogenic reactions instead of oncogenic genes, especially if we are thinking of oncogenesis in response to pharmacologic or environmental perturbations. Genetic defects then can be considered in terms of changes in activity, transcription, translation, or proteolysis.

**Table 4:**
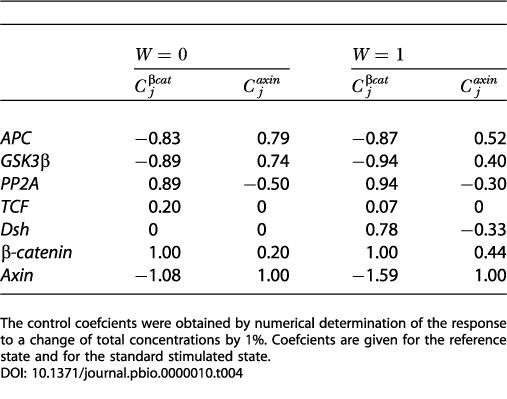
Concentration Control Coefficients for the Total Concentrations of β-Catenin and Axin Relative to Changes in the Concentrations of Pathway Components

The control coefficients were obtained by numerical determination of the response to a change of total concentrations by 1%. Coefficients are given for the reference state and for the standard stimulated state

Clearly, the robustness of a variable towards parameter changes is higher the lower the corresponding concentration control coefficient. To arrive at an estimation of the overall effects of parameter perturbations on the system as a whole, we consider first the standard deviation *σ* of the control coefficients from their mean value. According to the summation theorem, the mean value of all control coefficients for a given variable is zero. Thus, we get for the standard deviation for the control coefficients of β-catenin:

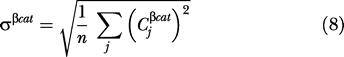
where the summation is performed over all reactions, including forward and backward steps of fast equilibria. High values of *σ* indicate that the given variable is on average very sensitive towards changes of rate constants. We propose to introduce a measure for the robustness *ρ* of a variable towards changes of all parameters in the following way:

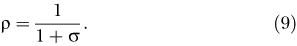



As *σ* may vary between zero and infinity, the range of *ρ* is confined to the interval 1≥ *ρ* ≥ 0. High values of *ρ* resulting from low *σ* values for the control coefficients indicate that the variable is robust against parameter perturbations. The standard deviations *σ* of the control coefficients and the *ρ* values for β-catenin and axin are presented in the last two rows of Table 3. Because many control coefficients are close to zero and the absolute values of the others hardly exceed unity, the *σ* values for β-catenin as well as for axin are rather small. Since all values for *σ* are lower than unity, a 1% change in a rate constant leads, on average, to a response of <1% in the overall level of β-catenin. The total concentration of axin is more robust against parameter perturbations than the total concentration of β-catenin, particularly in the standard stimulated state. A transition from the reference to the standard stimulated state results in a lower robustness for β-catenin and a higher robustness for axin.

## Discussion

Theory and quantitation are mutually dependent activities. It would seem unlikely that one would go to the trouble to measure detailed kinetic quantities without a specific model to test, and it is equally unlikely that realistic models can be constructed without the constraints of quantitative experimental data. Our intent in trying to reproduce a substantial part of the Wnt pathway in Xenopus egg extracts was to acquire the kind of detailed kinetic data required to build a realistic model. There are several unusual advantages to the extract system that contributed to this effort. The Xenopus egg extract is essentially neat cytoplasm; it reproduces the in vivo rate of β-catenin degradation and responds to known regulators as expected from in vivo experiments. Kinetic experiments with high time resolution are possible in this system, since a well-stirred extract is presumably synchronous in ways in which collections of cells may not be. In extracts it is possible to precisely set the level of components by depletion or addition. The direct output of the canonical Wnt pathway is an easily measured cytoplasmic event, the degradation of β-catenin. Thus, in this unusual system it is possible to acquire quantitative information about signaling pathways, not achievable in vivo. At the same time, these extracts have limitations. We have not considered the receptor events, and it is likely that reactions at the plasma membrane contribute to dynamic features. Also, our analysis is incomplete, as there are other components of Wnt signaling, such as casein kinase I*δ*, casein kinase I*ɛ*, and PAR1, as well as cross-talk from other pathways, that influence the behavior of the system. We have also oversimplified the multiple phosphorylation steps. We have assumed a simple interconversion of the phosphorylated and unphosphorylated complex of axin, APC, and GSK3β, whereas in reality multiple phosphorylation states exist within the complex; the states may be random or sequential. We simply do not have the information to provide a much more specific model of phosphorylation interconversions at this time, although the model could easily be extended. Finally, there is the question of what Wnt process we are studying. We are looking at events in the cytoplasm of unfertilized eggs. Though endowed with all of the core components of the Wnt pathway, the egg is, as far as we know, transcriptionally silent and not involved in Wnt signaling, though this system is active very soon in embryogenesis. Thus, there is no biological in vivo behavior with which to compare the in vitro behavior. Nevertheless, the basic core circuitry is intact and is presumably prepared for the early Wnt events in the embryo. All the properties of the egg extract system are very similar to that circuitry in vertebrate somatic cells.

To build a mathematical description of the Wnt signaling system, we started with the basic circuitry discerned from previous studies in Xenopus embryos and mammalian cells, whose similarity to the in vitro system we had already confirmed. We derived a system of differential equations that described the time-dependent variations of the system variables, i.e., the concentrations of the pathway components and their complexes. Parameters of the model are binding constants of proteins, rate constants of phosphorylations and dephosphorylations, rate constants of protein degradation, and rates of protein synthesis. Model reduction was achieved by considering conservation relations and by applying rapid equilibrium approximations for selected binding processes. Despite these simplifications, the model consists of a nonlinear system of differential equations whose solution requires the use of computers. Not all of these parameters were accessible to measurement. To circumvent this problem, we used as primary inputs not only kinetic parameters characterizing individual steps, but quantities that are more easily accessible from experiment, such as the overall flux of β-catenin degradation. This allowed us to derive rate constants as well as protein concentrations in a reference state, where there was no Wnt signal. This state serves as a starting point for predicting the system behavior during Wnt signaling as well as after experimental perturbations.

The basic model reproduced quantitatively the behavior of the reference state, including perturbations of this state achieved by varying the concentration of axin, GSK3β, and TCF. It also reproduced extensions of this to the signaling state. A wide variety of different sets of experimental data could be simulated by the same model, employing the same sets of kinetic parameters. We approached this process iteratively. For example, the early model did not include nonaxin-dependent degradation of β-catenin, but inclusion of this process improved the fit to the experimental data. More significantly, addition of this process had interesting biological implications, which we discuss.

In many ways, one of the most peculiar findings was the very low concentration of axin in the Xenopus extracts. Axin levels in other organisms may similarly be very low: *Drosophila* axin can be detected by Western blotting only following its immunoprecipitation (Willert et al. 1999). Although our theoretical and experimental studies have shown that axin is inhibitory at high concentrations, both indicate that axin is not present at the optimal concentration for the highest rate of β-catenin degradation. Therefore, axin levels are not set for optimality of β-catenin degradation, but are presumably optimized for some other purpose. Theoretically, axin levels must be held below the very sharp threshold of Dsh inhibition. Experimentally, these thresholds, which blunt Wnt signaling, are observed but are not as sharp as expected, and this may indicate some other compensatory effects. These thresholds would limit axin concentration to well below 1 nM if activated Dsh were constrained to concentrations of below 1 μM. Under these circumstances, we can expect that axin would never be found at concentrations approaching those of other Wnt pathway components (50–100 nM).

The low concentration of axin relative to other components (such as GSK3β, Dsh, and APC) has another design feature potentially very general and important for the modularity of metazoan signaling pathways. Axin is a critical node point for controlling β-catenin levels, but it also interacts with components shared with several other important pathways. The interaction of these components with axin fluctuates due to Wnt signals (reflecting changes in binding as well as changes in axin levels), yet because the concentration of axin is so low, there will be no appreciable change in the overall levels of GSK3β, Dsh, or APC (all these components important in other pathways would otherwise be driven to fluctuate). The very low axin concentration thus isolates the Wnt pathway from perturbing other systems, a simple mechanism to achieve modularity. Other scaffold proteins may serve similar functions in other pathways. These insights follow from a very simple measurement of axin concentration and suggest the utility of measuring the levels of signaling pathway components in different cell types and circumstances. Since quantitative and kinetic features may be important in defining modules, it suggests that qualitative circuit diagrams of signal transduction may overlook very important design features. Modularity within the Wnt pathway can be defined by an extension of the summation theorem of Heinrich and Rapoport (1974), which argues that the steady state of an entire pathway would have control coefficients that added to zero. When the Wnt pathway is broken down to several subpathways, we find that within these subpathways the control coefficients would sum to zero at steady state. While some of this subdivision is obvious (i.e., the kinase phosphatase module involving the phosporylation of APC and axin complexed to GSK3β), in other cases, such as the β-catenin module, it is much less obvious. Here the reactions include the phosphorylation of β-catenin in the APC/axin/GSK3β complex, the release and degradation of β-catenin, and the synthesis and nonaxin-dependent degradation of β-catenin. Balanced perturbation of these subpathways as a whole will not affect the overall flux of β-catenin degradation. It is not clear whether this concept of modularity might be extended usefully in two other directions: modularity in systems not at steady state, i.e., with transients, and estimates of linkage between pathways by some definition of nonzero summations expressing the degree of independence or modularity.

In addition to work by Kholodenko et al. (1997), this paper marks one of the first extensions of metabolic control theory to signal transduction. Metabolism and signal transduction seem very different, the former involving the transfer of mass and the latter the transfer of information. In addition, metabolic pathways generally involve dedicated components and the specificity of interaction of substrates and enzymes is very high. Signaling pathways share many components; interactions are often weak. Metabolism, which has had a long history of quantitative study, was a natural field for the development of control theory, and this theory has been successful in converting the specific information about the behavior of enzymes in a pathway to the overall behavior of metabolic circuits. Control coefficients are useful measures of the impact of a process or quantity on another. In its application to metabolism, it allowed us to dispose of erroneous concepts, such as the notion of a rate-determining step. In signal transduction, control coefficients might play a similar role. Here they can be used to indicate quantitively the effects of a particular reaction on some other property, such as flux through the pathway or concentration of another component. By this definition, certain rate constants, such as the phosphorylation and dephosphorylation of APC and axin, have a major influence on the levels of β-catenin, while others, such as the degradation rate of phosphorylated β-catenin, have little effect. The sign and magnitude of these control coefficients give some indication what gene products could be oncogenes or tumor suppressors. As shown in Table 4, by this criterion APC, GSK3β, and axin are potent tumor suppressors, whereas β-catenin is an oncogene. Dsh would be expected to exert only moderate oncogenic effects. Clearly the effects of certain gene products are dependent on context, including their rate of synthesis and steady-state concentration. As our understanding of pathways improve, the effect of mutation or pharmacologic inhibition could be estimated quantitatively using control coefficients. The differences between cell types and organisms could be exploited to better predict mutagenic and pharmacologic impact on signal propagation.

Despite considerable progress in identifying components of the Wnt pathway, many important mechanistic details are still lacking. In this analysis we have shown that Dsh seems to act to prevent the phosphorylation of the axin/APC complex, not the phosphorylation of β-catenin. Dsh (complexed to GBP) does not seem to be a general GSK3β inhibitor, like Li^+^, but rather is focused on the two scaffolding proteins. This was apparent from the biphasic nature of both the theoretical and experimental curves, which suggested that Dsh inhibited the rephosphorylation of axin/APC, but still allowed many cycles of β-catenin phosphorylation, ubiquitination, and degradation. This mechanism was further proven by a timing-of-addition experiment. It needs to be further confirmed and extended by looking specifically at individual phosphorylation sites on all the components of the complex. Another insight into the mechanisms of complex formation and control of β-catenin degradation concerns the inhibition of β-catenin degradation at concentrations of axin approaching those of other components. This suggests that axin binds APC, GSK3β, and β-catenin in random order. As discussed above, the axin concentration is limited by other factors; owing to the low concentration of axin, random binding is not likely ever to be a problem. The situation for APC seems very different. The concentration of APC is comparable to that of the other components. Overexpression studies show no inhibitory effects. These theoretical and experimental observations suggest that APC as a scaffold must be very different from axin as a scaffold. Most likely, APC binds components in an ordered manner.

Metabolic pathways are understandable in terms of the familiar logic of chemical synthesis; signal transduction pathways, by contrast, often do not seem to conform to simple design principles. It is not clear at all whether signaling pathways have been optimized for a specific function or instead whether they are remnants of some early and rather arbitrary evolutionary experiment, now embedded in other processes that are difficult to change. Systems analysis, along with experiment, offers some hope of uncovering latent principles of design. For example, the modeling of the Wnt pathway gave a theoretical insight into the function of axin degradation in Wnt signaling. The degree of axin instability dramatically affects the amplitude and duration of the β-catenin response to a transient Wnt signal. If axin turnover were designed to be slow, then β-catenin would rise slowly to a low amplitude and persist for many hours. In a system where axin turnover was more rapid, the amplitude could increase several-fold and would persist a shorter time. The duration and amplitude of the response are likely to be important factors in developmental systems, which may respond differently to different amplitudes and durations of a signal. In addition, some developmental processes occur with such rapidity that the same signal would be interpreted differently at different times, hence the need to quickly terminate a signal. The very different effects of transient and persistent signals in the same pathway have been studied in PC12 cells in the MAP kinase pathway activated by EGF or NGF (Marshall 1995). Finally, APC-dependent axin degradation stabilizes the Wnt pathway to variations in the APC concentration. Viewed from this perspective, the regulatory loop involving APC and axin degradation is an important design feature of the Wnt pathway.

Robustness has also been considered an important design principle for signaling processes (Alon et al. 1999), and control coefficients can be a good measure of this robustness. We present here a general measure of robustness of the entire pathway, using a measure that sums the variation in every individual reaction. Though it is generally thought that “robust” is good, the complement of robust is adaptability, and it may be that some aspects of a signaling pathway are designed to be responsive to changes in some parameters so that the same pathway can be used differently in different circumstances by altering key parameters. Since quantitative measures of the concentration or posttranslational modification of signaling proteins are rare in the literature, we have very little information on whether the organism varies certain key components to achieve different behavior of the signaling systems.

Another aspect of robustness is susceptibility to mutation or pharmacologic or environmental perturbation. The unexpected minimal phenotypes observed in numerous mouse knockout experiments have underscored our ignorance of the adaptable responses of organisms and in particular the adaptable nature of signaling pathways. One unexpected theoretical observation in this paper was the potential importance of the nonaxin-dependent degradation for β-catenin, under conditions where the APC levels are reduced. In our model, the nonaxin-dependent degradation contributed only a few percent to the overall flux of β-catenin degradation. Yet if the APC levels fall only 50%, the exact level of the alternative pathway made a large difference in the steady-state level of β-catenin. If the activity of the alternative pathways varied in different tissues, then this simple but largely silent effect could explain the tissue specificity of APC mutations. Similarly, variations in the alternative pathway might also explain some aspect of individual risk to loss of a single copy of the APC gene.

An experiment can better be judged by how many questions it raises than by how many it answers. The same may be said about theoretical analysis. Such analysis is always a work in progress, in that the experimental basis is continually changing to some degree. Some of the experimental changes, though significant mechanistically, may have little effect on the model and its interpretation. Some may require major revision. In the case of the Wnt pathway, the theoretical analysis and modeling have already raised several interesting questions of biological importance. They have already stimulated further experimentation. More than anything, the modeling has increased the urgency for obtaining accurate quantitative information about both steady-state and transient processes in Wnt signaling and for obtaining information about the differences in parameters in different tissues and in different organisms.

## Materials and Methods

### 

#### Egg extracts and degradation assays.

Xenopus egg extracts were prepared as described previously (Salic et al. 2000). Extracts were used either used immediately or stored at –80°C after being snap-frozen in liquid nitrogen. β-Catenin degradation assays were performed as described before (Salic et al. 2000).

#### Measurement of β-catenin synthesis.

Freshly prepared Xenopus egg extracts were either supplemented with 25 mM LiCl in Xenopus buffer (XB) or XB and an aliquot were withdrawn for β-catenin degradation assays. The free methionine pool in Xenopus embryos is approximately 90 μM, and based on this number, [^35^S]methionine was added to the extract to a give a final activity of 1,000 counts per picomole methionine. Extracts were incubated at 20°C, and aliquots were withdrawn at the indicated times for SDS-PAGE, trichloroacetic acid (TCA) precipitation, and β-catenin pull-downs. To assay protein synthesis in the extract, total methionine incorporation was measured by TCA precipitation. In brief, Xenopus extracts (2 μl), metabolically labeled with [^35^S]methionine as above, were diluted to 100 μl with PBS and supplemented with 2 μl of 2% deoxycholate and TCA to 5%. The reaction was pelleted at 20,000 × g for 10 min at 4°C. After washing with ice-cold acetone and air drying, the radioactivity of the pellet was measured in a scintillation counter.

To isolate metabolically labeled β-catenin from Xenopus extracts, we used His-tagged APCm3 cross-linked to Ultralink beads (Pierce, Woburn, Massachusetts, United States). Since phosphorylated APC has a much higher affinity for β-catenin, APCm3 beads were first phosphorylated with 300 nM His-tagged GSK3β in 25 mM HEPES (pH 7.7), 1 mM EDTA, 300 mM NaCl, 10 mM MgCl_2_, 1 mM DTT, and 1 mM ATP for 1 h at room temperature with shaking. The beads were washed three times with 25 mM HEPES (pH 7.7), 1 mM EDTA, 300 mM NaCl, 1% Tween, and 1 mM DTT and were then used to pull down β-catenin. Extracts (50 μl) labeled with [^35^S]methionine (see above) were diluted 5× with XB containing 1% Tween and protease inhibitors. The diluted extracts were incubated with phosphorylated APCm3 beads (20 μl) at 4°C for 2 h. The beads were washed and bound protein was eluted by boiling in SDS-PAGE loading buffer. Labeled β-catenin was detected following SDS-PAGE and autoradiography.

#### Measuring dissociation rates of phosphorylated/nonphosphorylated β-catenin from axin.

Radiolabeled β-catenin (5 μl) was phosphorylated using 300 nM His-tagged GSK3β and 100 nM maltose-binding protein (MBP)–axin in 20 μl of XB containing 10 mM ATP, 20 mM MgSO_4,_ and 50 mM NaCl. For the nonphorphorylated control, β-catenin was incubated as above, except that 50 mM LiCl was used instead of NaCl to inhibit GSK3β. The kinase reactions were incubated in a shaker for 30 min at 20°C and then added to 50 μl of MBP–axin, bound to amylose beads (1 mg of protein per milliliter of beads), and brought up to 250 μl with XB containing 50 mM LiCl. After phorsphorylated and nonphosphorylated β-catenin, respectively, bound to axin beads, the beads were washed three times with 500 μl of XB containing 50 mM LiCl. Dissociation of the bound β-catenin was initiated by adding 1 μM unlabeled recombinant β-catenin (His-tagged, from *Sf9* cells) and incubated at 20°C in a shaker. At the appropriate times, 5 μl aliquots of beads were quickly removed, filtered through Wizard minicolumns (Promega, Madison, Wisconsin, United States), and washed with ice-cold XB (3 ml). Proteins bound to beads were eluted from the minicolumns with 20 μl of hot sample buffer, followed by SDS-PAGE and autoradiography.

#### Recombinant proteins.

The expression and purification of all recombinant proteins have been previously described (Salic et al. 2000). Dsh was expressed as an MBP fusion in bacteria. His-tagged GSK3β, His-tagged Tcf3, His-tagged APCm3, and MBP–axin were expressed in *Sf9* cells.

## Supporting Information

Dataset S1 The Roles of APC and Axin Derived from Experimental and Theoretical Analysis of the Wnt Pathway (287 KB DOC).

Figure S1 Effect of Wnt Stimulation on the Concentrations of β-Catenin and Axin The curves represent steady-state concentrations of β-catenin (solid lines) and axin (broken lines) as functions of the strength *W* of Wnt stimulation. Curve a: free unphosphorylated β-catenin; curve b: free phosphorylated β-catenin; curve c: total β-catenin; curve d: total axin. All concentrations are scaled with respect to their values in the reference state. It is worth mentioning that in the model “without regulatory loop,” the steady-state concentration of free axin is determined by the condition *X*_12_ *=* **ν**_14_ */k*_15_, and is, therefore, independent of Wnt stimulation.
(2,954 KB TIFF).

Figure S2 Effects of the Amounts of Pathway Components on the Concentrations of β-Catenin and Axin This figure gives additional information with respect to the effects of Dsh, TCF, and GSK3β on the steady-state concentrations of total β-catenin (solid lines) and total axin (dashed lines) for the case of permanent Wnt-stimulation, *W* = 1. All concentrations and synthesis rates are scaled with respect to their values in the stimulated stationary state.
(3,472 KB TIFF).

Figure S3 Effects of Synthesis Rates on the Concentrations of β-catenin and Axin The curves represent steady-state values of total concentrations of β-catenin (solid lines) and axin (dashed lines), depending on the rates of synthesis of β-catenin and axin. All concentrations and synthesis rates are scaled with respect to their values in the stimulated stationary state.
(3,483 KB TIFF).

Table S1 Mathematical Notation for Model Variables as Subdivided into Independent and Dependent Variables (45 KB DOC).

Table S2 Complete List of Model Parameters of the Wnt Signal Transduction Model The rate constants marked with “#” play a role only in stimulated states where *W* ≠ 0. Note that some of the numerical values are given in a higher precision compared to Table 1.
(111 KB DOC).

## Abbreviations

APC: adenomatous polyposis coli
Dsh: Dishevelled
EDTA: ethylene diamine tetraacetic acid
GBP: glycogen synthase kinase-binding protein
GSK3β: glycogen synthase kinase 3β
MBP: maltose-binding protein
PP2A: protein phosphatase 2A
*Sf9*: *Spodoptera frugiperda*
TCA: trichloroacetic acid
TCF: T-cell factor
XB: Xenopus buffer.
