# Corrections: The Roles of APC and Axin Derived from Experimental and Theoretical Analysis of the Wnt Pathway

**DOI:** 10.1371/journal.pbio.0020089

**Published:** 2004-03-16

**In *PLoS Biology,* volume 1, issue 1:**

The Roles of APC and Axin Derived from Experimental and Theoretical Analysis of the Wnt Pathway

**Ethan Lee, Adrian Salic, Roland Krüger, Reinhart Heinrich, Marc W. Kirschner**

**DOI: 10.1371/journal.pbio.0000010**

Table 1: In the legend, the words *fluxes* and *flux* appeared without the *fl*.

Table 3: In the legend, the word *coefficients* appeared without the *fi*. In the table, some numbers in the section “Binding, dissociation” were marked with a ± sign that should have been a ∓.

Table 4: In the legend, the word *coefficients* appeared without the *fi*.

Please see the corrected legends and table below.

**Table 1.** Numeric Values of Input Quantities of the Model for the Reference State

The data are grouped into concentrations of pathway components, dissociation constants of protein complexes, concentration ratios, fluxes and flux ratios, and characteristic times of selected processes. Experimental evidence for these data is discussed in the text. From these data, the following rates and rate constants are calculated: **v**_12_ = 0.42 nM · min^−1^ (rate of β-catenin synthesis), **v**_14_ = 8.2 · 10^−5^ · nM · min^−1^ (rate of axin synthesis), *k*_4_ = 0.27 min^−1^, *k*_5_ = 0.13 min^−1^, *k*_6_ = 9.1 · 10^−2^ nM^−1^ · min^−1^, *k*_−6_ = 0.91 · nM^−1^ · min^−1^, *k*_9_ = 210 min^−1^, *k*_10_ = 210 min^−1^, *k*_11_ · 0.42 min^−1^, *k*_13_ = 2.6 · 10^−4^ min^−1^, *k*_15_ = 0.17 · min^−1^. See Table S2, found at http://dx.doi.org/10.1371/journal.pbio.0000010.t002, for more precise numbers used in the calculations.

**Bold:** Measured values, *Italics:* Estimated values.

DOI: 10.1371/journal.pbio.0000010.t001

[Table pbio-0020089-t001]

**Table 3 pbio-0020089-t001:**
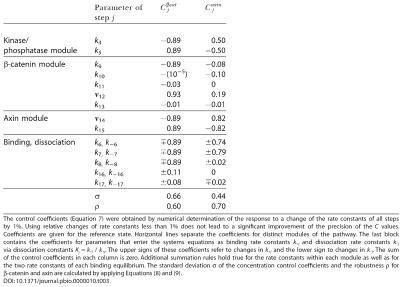
Control Coefficients for the Total Concentrations of β-Catenin and Axin and Parameters Quantifying the Sensitivity and the Robustness of the Wnt/β-Catenin Pathway

**Table 4.** Concentration Control Coefficients for the Total Concentrations of β-Catenin and Axin Relative to Changes in the Concentrations of Pathway Components

The control coefficients were obtained by numerical determination of the response to a change of total concentrations by 1%. Coefficients are given for the reference state and for the standard stimulated state.

DOI: 10.1371/journal.pbio.0000010.t004

The full text XML and HTML versions of the article have been corrected online. This correction note may be found at DOI: 10.1371/journal.pbio.0020089.

