# Supplementary material for: The Roles of APC and Axin Derived from Experimental and Theoretical Analysis of the Wnt Pathway
Source: PLoS Biol. 2003 Oct 13;1(1):e10. doi: 10.1371/journal.pbio.0000010 (PMC212691; doi:10.1371/journal.pbio.0000010)
Supplement: Dataset S1 — (287 KB DOC). [file pbio.0000010.sd001.doc]

**Supplemental Material**

### Modeling of the Wnt pathway: Mathematical Details

Here, we present a detailed mathematical description of the Wnt/$\beta$-catenin signal transduction pathway. This is made for the basic model where the two reactions contributing to a non-ordered binding of axin (binding of axin to $\beta$-catenin and GSK3, steps 18 and 19 in Figure 1) are omitted. The rates $v_{i}$ of the reactions *i* and the concentrations $X_{j}$ of the pathway components are numbered as indicated in Figure 1 and Table S1, respectively.

The time-dependent changes of the concentrations of these proteins and protein complexes are determined by the following system of differential equations:

$\frac{dX_{1}}{dt}=-v_{1}+v_{2}$ (A.1)

$\frac{dX_{2}}{dt}=v_{1}-v_{2}$ (A.2)

$\frac{dX_{3}}{dt}=v_{4}-v_{5}-v_{8}+v_{10}$ (A.3)

$\frac{dX_{4}}{dt}=-v_{3}-v_{4}+v_{5}+v_{6}$ (A.4)

$\frac{dX_{5}}{dt}=v_{3}-v_{6}$ (A.5)

$\frac{dX_{6}}{dt}=v_{3}-v_{6}+v_{7}$ (A.6)

$\frac{dX_{7}}{dt}=-v_{7}-v_{17}$ (A.7)

$\frac{dX_{8}}{dt}=v_{8}-v_{9}$ (A.8)

$\frac{dX_{9}}{dt}=v_{9}-v_{10}$ (A.9)

$\frac{dX_{10}}{dt}=v_{10}-v_{11}$ (A.10)

$\frac{dX_{11}}{dt}=$ $-v_{8}+v_{12}-v_{13}-v_{16}-v_{17}$ (A.11)

$\frac{dX_{12}}{dt}=-v_{7}+v_{14}-v_{15}$ (A.12)

$\frac{dX_{13}}{dt}=-v_{16}$ (A.13)

$\frac{dX_{14}}{dt}=v_{16}$ (A.14)

$\frac{dX_{15}}{dt}=v_{17}$ (A.15)

where the reaction rates on the right hand sides are functions of the system variables $X_{i}$ ($i$=1,…,15). Binding and dissociation processes of the scheme (reactions 6, 7, 8, 16, and 17) are described by the rate equations $v_{i}=k_{+i}X_{j}Y_{l}-k_{-i}\left(X_{j}.Y_{l}\right)$ where $X_{j}$ and $Y_{l}$ denote the free concentrations of the binding partners and $\left(X_{j}.Y_{l}\right)$ the concentrations of the complexes formed by the proteins. Syntheses of proteins (reactions 12 and 14) are described by constant rates $v_{i}=const$. Phosphorylations by the kinase (reactions 4 and 9), dephosphorylation by the phosphatase (reaction 5), as well as the irreversible release of phosphorylated $\beta$-catenin (reaction 10) are described by linear rate equations ($v_{i}=k_{i}X_{j}$). Activation of Dishevelled is described by $v_{1}=k_{1}X_{1}W$, where *W* denotes the normalized concentration the Wnt-ligand as described in the main text. Inactivation of Dishevelled is described by $v_{2}=k_{2}X_{2}$. Dishevelled mediated release of GSK3 is characterized by the rate equation $v_{3}=k_{3}X_{2}X_{4}$, where $X_{2}$ is considered to act as an activator of reaction 3, i.e. $X_{2}$ is not consumed in this reaction. Protein degradations (steps 11, 13, and 15) are described as first order processes with rates $v_{i}=k_{i}X_{j}$. Note that we refer here to the case "without regulatory loop" where the degradation rate of axin is independent of APC. Treatment of the case "with regulatory loop" where axin degradation depends on the APC concentration is straightforward, i.e. the kinetic equation $v_{15}=k_{15}^{'}X_{12}X_{7}/\left(K_{M}+X_{7}\right)$ should be used instead of $v_{15}=k_{15}X_{12}$.

The model described by the set of differential equations (A.1) - (A.15) can be simplified by considering conservation equations and by applying rapid equilibrium approximations to some of the binding reactions. This results in a subdivision of the complete set of system variables into 7 independent variables and 8 dependent variables. The latter variables are algebraic functions of the independent variables which are determined as solutions of differential equations. As shown below an appropriate choice for the independent variables is $X_{2}$, $X_{3}$, $X_{4}$, $X_{9}$, $X_{10}$, $X_{11}$, and $X_{12}$. For deriving the differential equation system for the independent variables the dependent variables $X_{1}$, $X_{5}$,$X_{6}$,$X_{7}$,$X_{8}$,$X_{13}$,$X_{14}$, and $X_{15}$ are eliminated from the full system (A.1) - (A.15) in a series of steps.

#### Conservation equations

The stoichiometry of the reaction scheme in Figure 1 implies the existence of 4 conservation equations, which are

$X_{1}+X_{2}=const.$ (A.16)

$X_{3}+X_{4}+X_{5}+X_{8}+X_{9}=const.$ (A.17)

$X_{3}+X_{4}+X_{6}+X_{7}+X_{8}+X_{9}+X_{15}=const.$ (A.18)

$X_{13}+X_{14}=const.$ (A.19)

i.e. the time derivatives of the given sums of concentrations equal zero. According to the reaction scheme these conservation quantities correspond to the total concentrations $Dsh^{0}$, $GSK3^{0}$, $APC^{0}$, and $TCF^{0}$.

##### Rapid equilibrium approximation

Rapid equilibrium approximations are made for all binding processes except for the binding of GSK3 to $\left(\text{APC}/\text{Axin}\right)$.

**Algebraic equations for dependent variables**

***Elimination of* $X_{5}$:** We apply Eq. (A.17) in a simplified form by taking into account, that the concentrations of GSK3 is very high compared to those of axin (cf. Table 2). Accordingly, we neglect in Eq. (A.17) the concentration of the axin containing complexes such that

$X_{5}=GSK^{0}$ (A.20)

which means that $X_{5}$ is not an independent variable but fixed by the conservation sum for GSK3.

***Elimination of* $X_{1}$:** This dependent variable is expressed by the independent variable $X_{2}$ by using the conservation equation (A.16)

$X_{1}=Dsh^{0}-X_{2}$. (A.21)

***Elimination of* $X_{7}$ *and*** $X_{15}$: These dependent variables can be expressed as functions of the independent variable $X_{11}$ by using the equilibrium condition

$\frac{X_{7}X_{11}}{X_{15}}=K_{17}$, (A.22)

and the conservation equation for APC (Eq. (A.18)). The latter equation is used in the simplified form

$APC^{0}=X_{7}+X_{15}$. (A.23)

This approximation is justified since all other terms on the left-hand side of Eq. (A.18) represent the concentration of complexes containing APC together with axin which occurs in very low concentration.

Combination of Eq. (A.22) and (A.23) yields

$X_{7}=\frac{K_{17}APC^{0}}{K_{17}+X_{11}}$, (A.24)

and

$X_{15}=\frac{X_{11}APC^{0}}{K_{17}+X_{11}}$. (A.25)

***Elimination of* $X_{13}$ *and*** $X_{14}$: These dependent variables can be expressed as functions of the variable $X_{11}$ by using the conservation equation for TCF (Eq. (A.19)) and the equilibrium condition

$\frac{X_{11}X_{13}}{X_{14}}=K_{16}$. (A.26)

This leads to

$X_{13}=\frac{K_{16}TCF^{0}}{K_{16}+X_{11}}$, (A.27)

and

$X_{14}=\frac{X_{11}TCF^{0}}{K_{16}+X_{11}}$. (A.28)

***Elimination of* $X_{8}$:** This dependent variable is eliminated by the equilibrium condition

$\frac{X_{3}X_{11}}{X_{8}}=K_{8}$ . (A.29)

This equation allows for a direct calculation of $X_{8}$ as a function of the independent variables $X_{3}$ and $X_{11}$.

***Elimination of* $X_{6}$:** This dependent variable can be expressed as functions of the independent variables $X_{11}$ and $X_{12}$ by using the equilibrium condition

$\frac{X_{7}X_{12}}{X_{6}}=K_{7}$ (A.30)

and by taking into account Eq. (A.24) for $X_{7}$. This leads to:

$X_{6}=\frac{K_{17}X_{12}APC^{0}}{K_{7}\left(K_{17}+X_{11}\right)}$. (A.31)

#### Differential equations for independent variables

***Differential equation for* $X_{2}$:** Substituting $X_{1}$ from Eq. (A.21) into the differential equation (A.2) yields

$\frac{dX_{2}}{dt}=k_{1} W \left(Dsh^{0}-X_{2}\right)-k_{2}X_{2}$ (A.32)

which can be solved for $X_{2}$ at a given extent of Wnt stimulation, either permanent, $W=const.$, or time dependent, $W=W\left(t\right)$.

***Differential equation for* $X_{9}$:** Introducing the rate equations into the right hand side of Eq. (A.9) yields

$\frac{dX_{9}}{dt}=k_{9}X_{8}-k_{10}X_{9}$, (A.33)

The dependent variable $X_{8}$ can be expressed as function of the independent variables $X_{3}$ and $X_{11}$ by using Eq. (A.29)which leads to:

$\frac{dX_{9}}{dt}=\frac{k_{9}X_{3}X_{11}}{K_{8}}-k_{10}X_{9}$. (A.34)

***Differential equation for* $X_{10}$:** Introducing the rate equations into the right hand side of Eq. (A.10) yields

$\frac{dX_{10}}{dt}=k_{10}X_{9}-k_{11}X_{10}$. (A.35)

***Differential equation for* $X_{4}$:** Introducing the rate equations into the right hand side of Eq. (A.4) yields:

$\frac{dX_{4}}{dt}=-k_{3}X_{2}X_{4}-k_{4}X_{4}+k_{5}X_{3}+k_{6}X_{5}X_{6}-k_{-6}X_{4}$. (A.36)

This equation contains the dependent variable $X_{6}$ which may be substituted using Eq. (A.31). From that it follows:

$\frac{dX_{4}}{dt}=-\left(k_{3}X_{2}+k_{4}+k_{-6}\right)X_{4}+k_{5}X_{3}+k_{6}X_{5}\frac{K_{17}X_{12}APC^{0}}{K_{7}\left(K_{17}+X_{11}\right)}$. (A.37)

***Differential equation for $X_{3}$,$X_{11}$, and* $X_{12}$:** The time-dependent variations of the concentration of these components are affected by rapid binding and dissociation processes as well as by slow steps. To derive equations for the variations on the slow time-scale, the rates $v_{7}$*, $v_{8}$, $v_{16}$,* and $v_{17}$ of the fast binding processes must be eliminated. This is achieved by appropriate linear combination of differential equations (for details of the method: Heinrich & Schuster, The Regulation of Cellular Systems, Chapman and Hall, New York, 1996):

$\frac{d\left(X_{6}+X_{12}\right)}{dt}=v_{3}-v_{6}+v_{14}-v_{15}$. (A.38)

$\frac{d\left(X_{3}+X_{8}\right)}{dt}$ $=v_{4}-v_{5}-v_{9}+v_{10}$, (A.39)

$\frac{d\left(X_{8}+X_{11}+X_{14}+X_{15}\right)}{dt}=-v_{9}+v_{12}-v_{13}$ (A.40)

These equations still contain dependent variables which must be eliminated.

$X_{6}$ on the left-hand side of Eq. (A.38) is eliminated on the basis of Eq. (A.31), which describes $X_{6}$ as a function of $X_{11}$, and $X_{12}$*.* In this way, the time variation of $X_{6}$ may be expressed as follows

$\frac{dX_{6}}{dt}=\frac{\partial X_{6}}{\partial X_{11}}\frac{dX_{11}}{dt}+\frac{\partial X_{6}}{\partial X_{12}}\frac{dX_{12}}{dt}$. (A.41)

On the right-hand side of Eq. (A.38) the term for step 6 contains the dependent variables $X_{5}$ and $X_{6}$ which can be eliminated using Eqs. (A.20) and (A.31). In this way, Eq. (A.38) is transformed into

$$\frac{dX_{12}}{dt}\left(1+\frac{APC^{0}K_{17}}{K_{7}\left(K_{17}+X_{11}\right)}\right)-\frac{dX_{11}}{dt}\frac{APC^{0}K_{17}X_{12}}{K_{7}\left(K_{17}+X_{11}\right)^{2}}=$$

$=k_{3}X_{2}X_{4}-k_{6}\frac{GSK^{0}APC^{0}K_{17}X_{12}}{K_{7}\left(K_{17}+X_{11}\right)}+k_{-6}X_{4}+v_{14}-k_{15}X_{12}$ . (A.42)

In Eq. (A.39) the dependent variable$X_{8}$, which appears on the left-hand side and the right-hand side is eliminated on the basis of Eq. (A.29), which describes $X_{8}$ as a function of $X_{3}$ and $X_{11}$*.* In this way, Eq. (A.39) is transformed into

$k_{4}X_{4}-k_{5}X_{3}-\frac{k_{9}X_{3}X_{11}}{K_{8}}+k_{10}X_{9}$. (A.43)

On the left-hand side of Eq. (A.40) the time derivatives of the dependent variables $X_{8}$, $X_{14}$, and $X_{15}$ may be expressed by time-derivatives of the independent variables $X_{11}$, and $X_{3}$ (cf. Eqs. (A.29), (A.28), and (A.25))*.* The kinetic equation for step 9 contain the dependent variable $X_{8}$, which can be eliminated using Eq. (A.29). In this way, Eq. (A.40) is transformed into

$\frac{dX_{11}}{dt}\left(1+\frac{X_{3}}{K_{8}}+\frac{TCF^{0}K_{16}}{\left(K_{16}+X_{11}\right)^{2}}+\frac{APC^{0}K_{17}}{\left(K_{17}+X_{11}\right)^{2}}\right)+\frac{X_{11}}{K_{8}}\frac{dX_{3}}{dt}=v_{12}-\left(\frac{k_{9}X_{3}}{K_{8}}+k_{13}\right)X_{11}$ (A.44)

The 7 independent variables are obtained by solving the system of differential equations (A.32), (A.34), (A.35), (A.37), (A.42), (A.43), and (A.44). The 8 dependent variables can be calculated using the algebraic equations (A.20), (A.21), (A.24), (A.25), (A.27), (A.28), (A.29), and (A.31). In Table S2 all model parameters are listed which should be used for numerical integration.
