# Supplementary figures and images for: The Roles of APC and Axin Derived from Experimental and Theoretical Analysis of the Wnt Pathway
Source: PLoS Biol. 2003 Oct 13;1(1):e10. doi: 10.1371/journal.pbio.0000010 (PMC212691; doi:10.1371/journal.pbio.0000010)

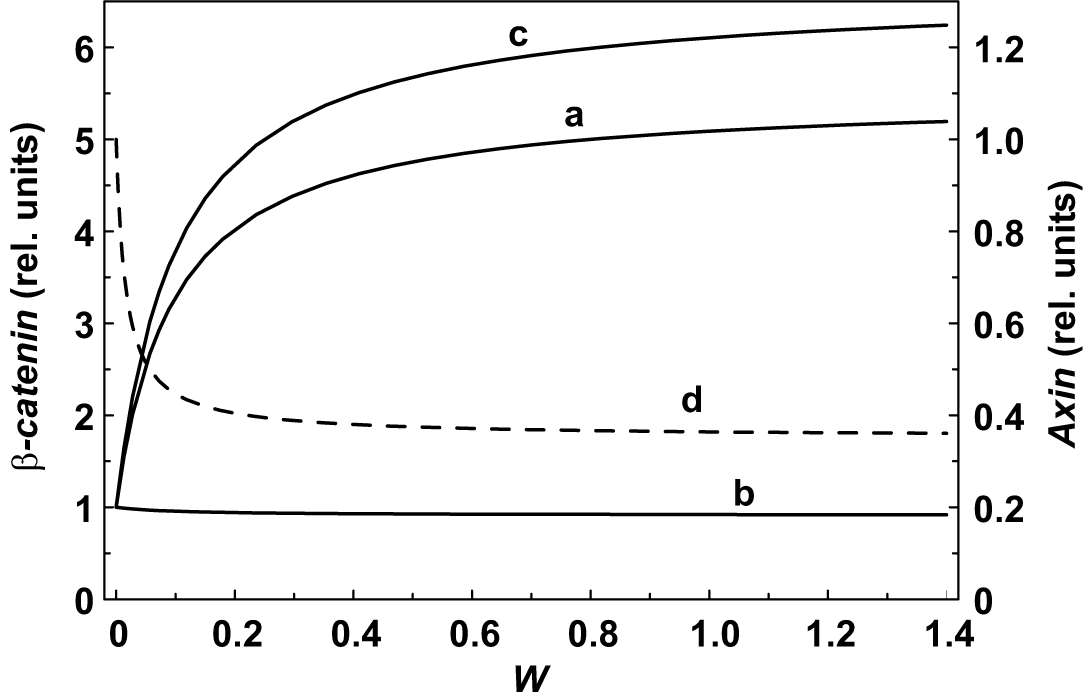

Supplement: Figure S1 — The curves represent steady-state concentrations of β-catenin (solid lines) and axin (broken lines) as functions of the strength W of Wnt stimulation. Curve a: free unphosphorylated β-catenin; curve b: free phosphorylated β-catenin; curve c: total β-catenin; curve d: total axin. All concentrations are scaled with respect to their values in the reference state. It is worth mentioning that in the model “without regulatory loop,” the steady-state concentration of free axin is determined by the condition X 12 = ν 14 /k 15, and is, therefore, independent of Wnt stimulation. (2,954 KB TIFF). [file pbio.0000010.sg001.tif]

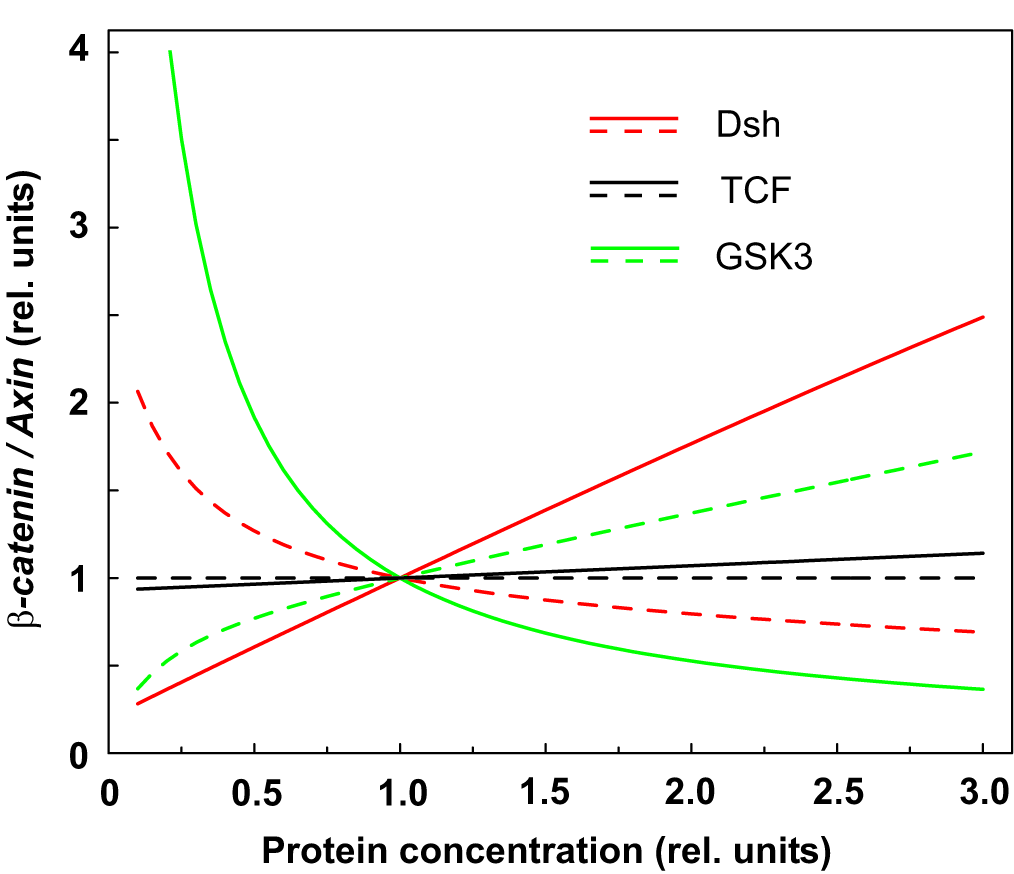

Supplement: Figure S2 — This figure gives additional information with respect to the effects of Dsh, TCF, and GSK3β on the steady-state concentrations of total β-catenin (solid lines) and total axin (dashed lines) for the case of permanent Wnt-stimulation, W = 1. All concentrations and synthesis rates are scaled with respect to their values in the stimulated stationary state. (3,472 KB TIFF). [file pbio.0000010.sg002.tif]

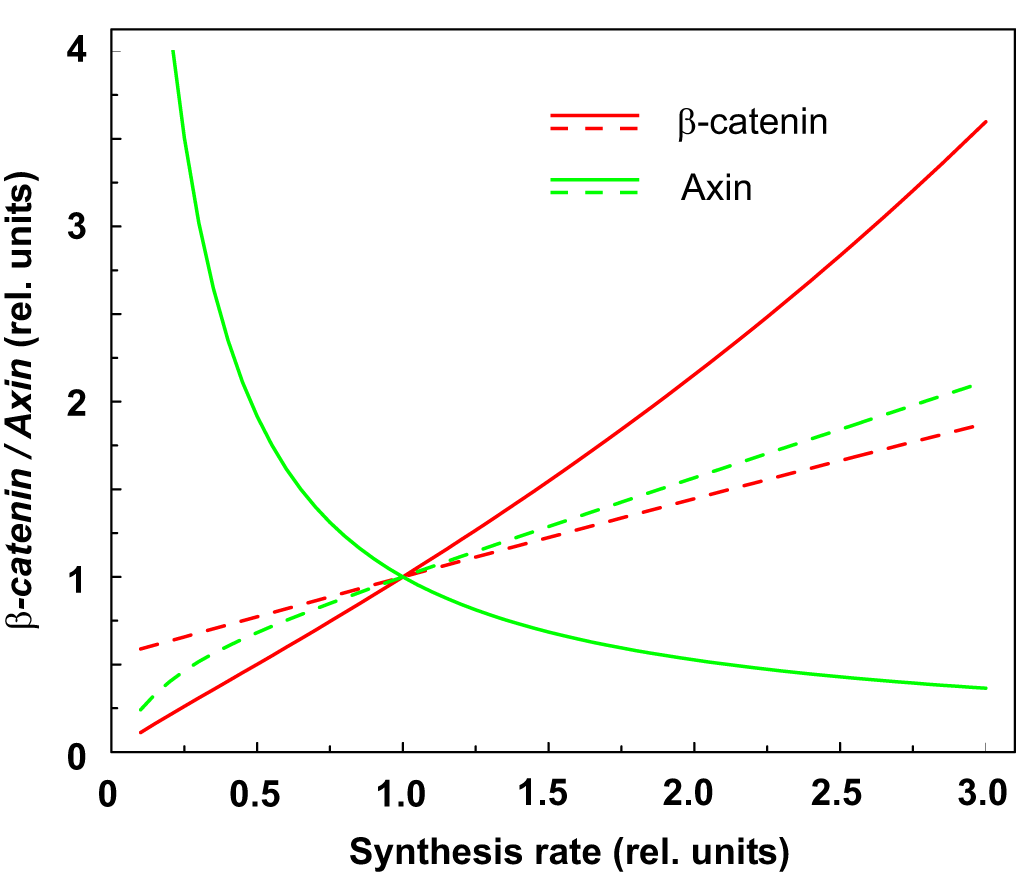

Supplement: Figure S3 — The curves represent steady-state values of total concentrations of β-catenin (solid lines) and axin (dashed lines), depending on the rates of synthesis of β-catenin and axin. All concentrations and synthesis rates are scaled with respect to their values in the stimulated stationary state. (3,483 KB TIFF). [file pbio.0000010.sg003.tif]
