# Supplementary material for: The Roles of APC and Axin Derived from Experimental and Theoretical Analysis of the Wnt Pathway
Source: PLoS Biol. 2003 Oct 13;1(1):e10. doi: 10.1371/journal.pbio.0000010 (PMC212691; doi:10.1371/journal.pbio.0000010)
Supplement: Table S1 — (45 KB DOC). [file pbio.0000010.st001.doc]

#### Table S1. Mathematical Notation for Model Variables as Subdivided into Independent and Dependent Variables

| Concentration | Component |
| --- | --- |
| *independent variables* | |
| $X_{2}$ | Dsh_a_ |
| $X_{3}$ | (APC*/axin*/GSK3) |
| $X_{4}$ | (APC/axin/GSK3) |
| $X_{9}$ | (β-catenin*/APC*/axin*/GSK3) |
| $X_{10}$ | β-catenin* |
| $X_{11}$ | β-catenin |
| $X_{12}$ | Axin |
| *dependent variables* | |
| $X_{1}$ | Dsh_i_ |
| $X_{5}$ | GSK3 |
| $X_{6}$ | (APC/axin) |
| $X_{7}$ | APC |
| $X_{8}$ | (β-catenin/APC*/axin*/GSK3) |
| $X_{13}$ | TCF |
| $X_{14}$ | (β-catenin/TCF) |
| $X_{15}$ | (β-catenin/APC) |
