# Supplementary material for: The Roles of APC and Axin Derived from Experimental and Theoretical Analysis of the Wnt Pathway
Source: PLoS Biol. 2003 Oct 13;1(1):e10. doi: 10.1371/journal.pbio.0000010 (PMC212691; doi:10.1371/journal.pbio.0000010)
Supplement: Table S2 — The rate constants marked with “#” play a role only in stimulated states where W ≠ 0. Note that some of the numerical values are given in a higher precision compared to Table 1. (111 KB DOC). [file pbio.0000010.st002.doc]

**Table S2.** Complete List of Model Parameters of the Wnt-Signal Transduction Model

The rate constants marked with (#) play a role only in stimulated states where $W\neq0$. Note that some of the numerical values are given in a higher precision compared to Table S1.

| Parameter | Value | |
| --- | --- | --- |
| *Conservation quantities* |  |  |
| $Dsh^{0}$ | 100 | nM |
| $APC^{0}$ | 100 | nM |
| $TCF^{0}$ | 15 | nM |
| $GSK^{0}$ | 50 | nM |
| *Dissociation constants* |  |  |
| $K_{7}$ | 50 | nM |
| $K_{8}$ | 120 | nM |
| $K_{16}$ | 30 | nM |
| $K_{17}$ | 1200 | nM |
| *Rate constants* |  |  |
| $k_{1}$ (#) | $0.182$ | $\text{min}^{-1}$ |
| $k_{2}$ (#) | $1.82\cdot10^{-2}$ | $\text{min}^{-1}$ |
| $k_{3}$ (#) | $5.00\cdot10^{-2}$ | $\text{nmol}^{-1} \text{min}^{-1}$ |
| $k_{4}$ | $0.267$ | $\text{min}^{-1}$ |
| $k_{5}$ | $0.133$ | $\text{min}^{-1}$ |
| $k_{6}$ | $9.09\cdot10^{-2}$ | $\text{nmol}^{-1} \text{min}^{-1}$ |
| $k_{-6}$ | $0.909$ | $\min^{-1}$ |
| $k_{9}$ | $206$ | $\min^{-1}$ |
| $k_{10}$ | $206$ | $\min^{-1}$ |
| $k_{11}$ | $0.417$ | $\min^{-1}$ |
| $k_{13}$ | $2.57\cdot10^{-4}$ | $\min^{-1}$ |
| $k_{15}$ | $0.167$ | $\min^{-1}$ |
| *Synthesis fluxes* |  |  |
| $v_{12}$ | $0.423$ | $\text{nmol} \text{min}^{-1}$ |
| $v_{14}$ | $8.22\cdot10^{-5}$ | $\text{nmol} \text{min}^{-1}$ |
